# AKT-driven epithelial-mesenchymal transition is affected by copper bioavailability in HER2 negative breast cancer cells via a LOXL2-independent mechanism

**DOI:** 10.1007/s13402-022-00738-w

**Published:** 2022-12-01

**Authors:** Alessandra Vitaliti, Ilenia Roccatani, Egidio Iorio, Nunzio Perta, Angelo Gismondi, Mattea Chirico, Maria Elena Pisanu, Daniele Di Marino, Antonella Canini, Anastasia De Luca, Luisa Rossi

**Affiliations:** 1 Department of Biology, University of Rome “Tor Vergata”, Via Della Ricerca Scientifica 1, 00133 Rome, Italy; 2 Core Facilities High Resolution NMR Unit, Istituto Superiore Di Sanità, 00161 Rome, Italy; 3 Department of Life and Environmental Sciences, Polytechnic University of Marche, Via Brecce Bianche, 60131 Ancona, Italy; 4 PhD program in Cellular and Molecular Biology, Department of Biology, University of Rome “Tor Vergata”, Via della Ricerca Scientifica 1, 00133 Rome, Italy

**Keywords:** Breast cancer, Epithelial to mesenchymal transition, Copper, TRIEN, AKT, TGFβ, LOXL2, HER2

## Abstract

**Background:**

The main mechanism underlying cancer dissemination is the epithelial to mesenchymal transition (EMT). This process is orchestrated by cytokines like TGFβ, involving “non-canonical” AKT- or STAT3-driven pathways. Recently, the alteration of copper homeostasis seems involved in the onset and progression of cancer.

**Methods:**

We expose different breast cancer cell lines, including two triple negative (TNBC) ones, an HER2 enriched and one cell line representative of the Luminal A molecular subtype, to short- or long-term copper-chelation by triethylenetetramine (TRIEN). We analyse changes in the expression of EMT markers (E-cadherin, fibronectin, vimentin and αSMA), in the levels and activity of extracellular matrix components (LOXL2, fibronectin and MMP2/9) and of copper homeostasis markers by Western blot analyses, immunofluorescence, enzyme activity assays and RT-qPCR. Boyden Chamber and wound healing assays revealed the impact of copper chelation on cell migration. Additionally, we explored whether perturbation of copper homeostasis affects EMT prompted by TGFβ. Metabolomic and lipidomic analyses were applied to search the effects of copper chelation on the metabolism of breast cancer cells. Finally, bioinformatics analysis of data on breast cancer patients obtained from different databases was employed to correlate changes in kinases and copper markers with patients’ survival.

**Results:**

Remarkably, only HER2 negative breast cancer cells differently responded to short- or long-term exposure to TRIEN, initially becoming more aggressive but, upon prolonged exposure, retrieving epithelial features, reducing their invasiveness. This phenomenon may be related to the different impact of the short and prolonged activation of the AKT kinase and to the repression of STAT3 signalling. Bioinformatics analyses confirmed the positive correlation of breast cancer patients’ survival with AKT activation and up-regulation of CCS. Eventually, metabolomics studies demonstrate a prevalence of glycolysis over mitochondrial energetic metabolism and of lipidome changes in TNBC cells upon TRIEN treatment.

**Conclusions:**

We provide evidence of a pivotal role of copper in AKT-driven EMT activation, acting independently of HER2 in TNBC cells and via a profound change in their metabolism. Our results support the use of copper-chelators as an adjuvant therapeutic strategy for TNBC.

**Supplementary Information:**

The online version contains supplementary material available at 10.1007/s13402-022-00738-w.

## Background

Breast cancer is the second most common cancer worldwide, after lung cancer. It is a highly heterogeneous malignancy, the classification of which is based on histopathological and molecular features.

Regarding molecular classification, breast cancer comprises the following subtypes: Luminal A, expressing both the estrogen and progesterone receptor (ER + and PR +), Luminal B that in addition to ER and PR expresses a low level of the human epidermal receptor 2 (HER2) (ER + , PR + , HER2low), and the HER2-enriched subtype (ER-, PR-, HER2 +). The latter can be treated with therapy targeting HER2. The most aggressive and highly heterogeneous of all subtypes is the Triple Negative Breast Cancer (TNBC), lacking the expression of ER, PR and also of HER2, thus poorly targetable with chemotherapy, and characterized by a great metastatic potential and poor prognosis [1].

The mechanisms underlying cancer dissemination are the epithelial to mesenchymal transition (EMT), the extracellular matrix (ECM) remodeling and angiogenesis [2].In particular, during EMT, a dynamic and pleiotropic program, epithelial cancer cells lose their baso-apical polarization and, following the disruptions of tight and adherens junctions acquire a mesenchymal phenotype, gaining a spindle shape morphology. The fulfillment of this process confers to cells the ability to detach from the primary tumor mass becoming highly metastatic. The crucial step of EMT is the expression of EMT transcription factors (EMT-TFs) SNAI1, SNAI2, TWIST1/2 and ZEB1. They are in charge of the downregulation of the epithelial marker E-cadherin (Epithelial cadherin) as well as of the upregulation of N-cadherin (Neural cadherin) (a mechanism also known as the “cadherin switch” [3]) and of the mesenchymal proteins fibronectin, vimentin and α smooth muscle actin (αSMA) [4].

The expression of the EMT-TFs can occur because of a variety of molecular stimuli, such as the transforming growth factor beta (TGFβ), the bone marrow protein (BMP), Wnt / β-catenin, NOTCH, Shh and the activation of receptor tyrosine kinases (RTKs). The principal one is TGFβ, which binding to its type I or II receptors (TGFβRI and TGFβRII) induces their autophosphorylation, thus activating several (canonical or non-canonical) transduction pathways leading to EMT. The “canonical” pathway involves SMAD (Small Mothers Against Decapentaplegic) homologous proteins 2, 3 and 4. In detail, the phosphorylation of TGFβRI results in the oligomerization and phosphorylation of SMAD2 and SMAD3 followed by the recruitment of SMAD4, with the formation of a ternary complex. On the other hand, the “non-canonical” pathway of TGFβ, activated upon engagement of the RTKs, contributes to the activation of EMT-TFs by the Phosphatidylinositol 3-kinase (PI3K)/RAC-alpha serine/threonine-protein kinase (AKT) and MEK/ERK axes[4, 5]. Additionally, in TNBC it has been recently shown the pivotal role of glycogen synthase kinase 3β (GSK3β) in the modulation of EMT [6]. The signaling cascade of GSK3β interconnects with that of PI3K/AKT: AKT activation results in the phosphorylation and subsequent inactivation of GSK3β leading to the decrease of the ubiquitination of the transcription factor SNAI1 and thus in an increase of its level.

Another “non-canonical” TGFβ pathway, pursued upon engagement of RTKs, involves the activation of the Janus kinase (JAK)/Signal Transducer and Activator of Transcription 3 (STAT3) axis. STAT3, upon phosphorylation, translocates to the nucleus to regulate the transcription of several genes associated with cancer progression [7].

A key step of EMT is also the remodeling of the ECM, involving the activation of matrix metalloproteinases (MMPs). Crucial is the role of MMPs in the promotion of cell migration. Indeed, the activity of MMP9 and MMP3 is required for the shedding of the ectodomain of E-cadherin and thus for disassembling adherens junctions [8–10].

In the latest years, many reports demonstrate that the homeostasis of the essential transition metal copper (Cu) is intrinsically involved in breast cancer growth and spreading. Brain tumor cells show much higher Cu levels than corresponding non-cancerous cells. Elevated Cu concentrations have been reported in tumor tissue and serum from both animal models and patients with breast, lung, thyroid, cervix, stomach and prostate cancers [11, 12]. A growing body of evidence supports the involvement of Cu-proteins/enzymes as fundamental for the onset and progression of cancer via EMT and ECM remodeling. Two Cu-dependent proteins are required for ECM remodelling: LOXL2, belonging to the lysil oxidase (LOX) family proteins [13] and the Mediator of ErbB2-driven cell Motility 1 (MEMO1) [14]. LOXL2, after glycosylation, is secreted in the ECM, promoting collagen I fiber alignment and crosslinking [15], whilst MEMO1, besides activating PI3K/AKT axis leading to EMT activation, enhances the anchorage independent properties of TNBC cell lines and the metastatic potential [14, 16].

Furthermore, other findings support the involvement of additional Cu-linked proteins in cancer. Indeed, Cu homeostasis is a complex and finely tuned process, which must be functional at its best to ensure cell survival. A net of specific protein transporters and chaperones, which discretely distributes the metal to Cu-requiring enzymes and excrete useless noxious Cu from the cell, orchestrates it. It has been demonstrated the up-regulation in breast cancer of the Cu-transporter CTR1, required for the intracellular import of Cu, and of the copper P-type ATPase pumps ATP7A and B, located in the trans-Golgi network; they are involved in delivering of Cu the Cu-proteins with an extracellular destiny (e.g. LOX) and, by trafficking to the plasma membrane, to the release of excess of Cu outside the cells. Furthermore, the cytosolic Cu chaperone Atox1, which loads ATPases with Cu, is up-regulated in breast cancer [17], and it is also involved in angiogenesis, acting as a transcription factor for NADPH oxidase, leading to neo-vascularization [18]. In particular, in TNBC, Atox1/ATP7A/LOX axis prompts cell migration [19, 20]. Moreover, the up-regulation of other Cu-chaperones involved in Cu distribution to mitochondria and cytochrome c oxidase, i.e. COX17 and SCO2, was reported in breast cancer [17].

Of note, Cu binds and modulates also the activity of some members of the MAPK family and of the kinases surveilling the autophagy pathways: MEK1/2 (MAP2K1, Mitogen Activated Protein Kinase Kinase) and Unc-51 like kinase 1/2 (ULK1/2), respectively [12, 21, 22]. Cu also controls the activity of the E3 ubiquitin ligase XIAP1, which inhibits apoptosis and in turn regulates TGFβ signaling [2].

Based on all this evidence, Phase II clinical trials aimed to the reduction of Cu availability, achieved by the use of Cu chelating drugs, have been linked to the reduction of the metastatic progression in TNBC [23], as well as in other types of cancer [24].

However, to date, the role of Cu transporters/proteins in breast cancer is still unclear, although they may represent possible therapeutic targets, in particular for TNBC. Therefore, the present work aimed to gain more insight into the relationship between Cu homeostasis and the expression of EMT markers during TGFβ-induced EMT. We reduced the bioavailability of Cu by treating cultured breast cancer cells with a known Cu-chelator, triethylenetetramine (TRIEN), a consolidate drug used for the therapy of the genetic Cu overload disease (Wilson's disease), and more recently in cancer clinical trials [24]. As cell models, we used several breast cancer cell lines, including two TNBCs (i.e., MDA-MB-231 and SUM159), a HER2-enriched breast cancer cell line (SK-BR-3), and a cell line representative of the molecular subtype Luminal A (i.e., T47D).

Our data provide evidence of a pivotal role of Cu in the activation of the “non-canonical” AKT driven EMT induced by TGFβ, acting in a HER2-independent manner and through the implementation of a profound change in TNBC cells metabolism. The role of deregulation of Cu homeostasis in the modulation of EMT offers an additional opportunity to identify new therapeutic strategies to counteract breast cancer dissemination.

## Methods

### Cell culture and treatments

The human triple negative breast cancer (TNBC) cell line MDA-MB-231, the HER2-overexpressing SK-BR-3 cell line and the HER2^−^ PR^+^ER^+^ T47D cell line, were obtained from the American Type Culture Collection (ATCC, Manassas, VA, USA, cat. no. HTB-26™, HTB-30™and HTB-133™, respectively). The TNBC cell line SUM159 was kindly provided by Dr. Alessio Ottaviani, Department of Biology, University of Rome “Tor Vergata”. Cells were grown in high glucose Dulbecco’s modified medium (DMEM), supplemented with 10% heat-inactivated fetal bovine serum (FBS), 2 mM glutamine, 100 U/mL penicillin and 100 µg/mL streptomycin (EuroClone, Milano, Italy) (complete medium), and incubated at 37 °C, in a humidified atmosphere with 5% CO_2_.

For Cu depletion, MDA-MB-231, SK-BR-3, T47D (0.3 × 10^4^ cells/cm^2^) and SUM159 (0.1 × 10^4^ cells/cm^2^) were seeded in 6 wells plates. The day after plating, 125 µM Cu-chelator triethylenetetramine, TRIEN (Merck Life Science, Srl, Milano, Italy), was added to the cell culture medium. Cells were harvested and analyzed after 24 and 48 h. In the 6 days TRIEN treatment protocol, 125 µM TRIEN was added to the cell culture medium the day after plating and maintained for 3 days, at which time cells reached confluence (end of the first passage in culture in the presence of TRIEN). Afterwards, cells were trypsinized and plated again in 6 well plates with the same concentration of TRIEN. In some experiments, alternatively to TRIEN, tetrathiomolybdate (TTM, Merck Life Science, Srl, Milano, Italy), another Cu-chelator, was added to the cells at the concentration of 5 µM.

In order to restore Cu bioavailability after its depletion, in control experiments, the cell culture medium was replaced with one containing 100 μM copper sulfate (CuSO_4_, Merck Life Science, Srl, Milano, Italy) and kept for up to 3 h before analysis.

To induce EMT, cells were seeded in 6-well plates at the above reported cell densities and incubated for the next 24 h in DMEM containing only 1% FBS (in order to achieve cell starvation and cell cycle synchronization), before adding 10 ng/ml of Transforming Growth Factor β (TGFβ, eBIOSCIENCE, San Diego, CA, USA) for an additional 24 h. In some experiments, TGFβ was added to cells already treated with TRIEN for 6 days and TRIEN was maintained in the culture medium together with TGFβ for additional 24 h (for a total of 7 days of treatment with TRIEN).

### Cell viability assessment by MTS assay

Cells viability was measured by the capability of cell dehydrogenases to reduce MTS (3- [4,5-dimethylthiazol-2-yl] -5- [3-carboxymethylphenyl] -2-[4-sulphophenyl] -2H-tetrazolium inner salt) (Promega, Madison, USA) to a formazan chromogenic compound. Cells were plated in 96-well plates and, at the end of treatment, the culture medium in each well was replaced with a medium containing MTS. The change in color was recorded at 492 nm, by a microplate reader.

### Preparation of samples and Western blot analysis

After treatments, cells were harvested, washed in PBS, and suspended in lysis buffer containing 10 mM Tris–HCl (pH 7.4), 1 mM EDTA, 1 mM EGTA, 1% NP-40, 30 mM NaCl, supplemented with protease inhibitor cocktail (Merck Life Science, Milano, Srl, Italy). After 20 min incubation on ice, the samples were centrifuged at 1000 × g for 20 min, at 4 °C. The protein concentration of the suspended pellets was determined using the Lowry colorimetric assay (DC™ ProteinAssay, BioRad, Hercules, CA, USA). The samples were diluted in Laemmli Buffer 2X, plus 5% β-mercaptoethanol and denatured at 95° C for 5 min.

For the evaluation of extracellular LOXL2 and fibronectin an aliquot of cell medium was centrifuged at 2000 × g for 5 min, supernatant supplemented with a saturated solution of ammonium sulphate, centrifuged and the pellet suspended in 100 μl of ddH_2_O and 20 μl Laemmli Buffer 5 × was added and the samples treated as above.

Proteins (20 μg or 25 µl for sample from cell medium) were separated on 8 or 12% SDS–polyacrylamide gel and transferred to an Immobilon-PVDF transfer membrane (Millipore, Billerica, MA, USA). Table 1 reports primary antibodies used for immunodetection, as well as their dilutions. Anti-rabbit or anti-mouse secondary antibodies (Cell Signaling, Danvers, MA, USA) were revealed with the ECL (ECL Prime Western Blotting Reagent, Cytiva Europe GmbH, Freiburg, Germany) by the ImageQuant LAS 4000 (Fuji Film, Tokyo, Japan). Densitometric analyses were performed through the ImageJ software (NIH, Bethesda, MD, USA). Vinculin or actin were used as loading control for antigen protein to be evaluated in cell extract, while Ponceau S staining (Sigma-Aldrich, St. Louis, MO, USA) was used as loading control for the extracellular LOXL2.

**Table 1:** Primary antibodies used for Western blot analysis

Primary Antibody	Type	Company	Diluition
E-cadherin	Mouse	BD Transduction Laboratories #610,181	1:1000
Fibronectin	Rabbit	Merck Life Science S.r.l #F3648	1:5000
Vimentin	Mouse	Merck Life Science S.r.l #V6389	1:5000
αSMA	Mouse	Abcam #ab7817	1:1000
ATP7A	Mouse	Santa Cruz Biotechnology #376,467	1:1000
ATP7B	Mouse	Santa Cruz Biotechnology #373,964	1:1000
CCS	Rabbit	Santa Cruz Biotechnology #517,412	1:1000
Complex IV subunit II	Mouse	Molecular Probes #A-6404	1:1000
MEMO1	Mouse	Santa Cruz Biotechnology #517,412	1:1000
LOXL2	Mouse	Santa Cruz Biotechnology #293,427	1:1000
SMAD2	Rabbit	CellSignaling #3122	1:1000
Phospho-SMAD2 (Ser465/467)	Rabbit	CellSignaling #3108	1:1000
SMAD3	Rabbit	CellSignaling #9513	1:1000
Phospho-SMAD3 (Ser423/425)	Rabbit	CellSignaling #9520	1:1000
p44/42MAPK (Erk1/2)	Rabbit	CellSignaling #4695	1:1000
Phospho-p44/42 MAPK(Erk1/2) (Thr202/Tyr204)	Rabbit	CellSignaling #9101	1:5000
AKT	Rabbit	CellSignaling #4691	1:1000
Phospho-Akt (Ser473)	Rabbit	CellSignaling #4058	1:5000
Phospho-STAT3 (pSTAT3)	Rabbit	CellSignaling #9145	1:1000
SNAI1	Mouse	Santa Cruz Biotechnology #393,172	1:1000
GAPDH	Rabbit	Merck Life Science S.r.l #G9545	1:5000
Actin	Rabbit	Merck Life Science S.r.l #A2066	1:5000
Vinculin	Mouse	Santa Cruz Biotechnology #25,336	1:8000

### Immunofluorescence analysis

Cells were grown on coverslips and treated as previously described (see Cell culture and treatments paragraph). After treatments, cells were washed three times in PBS and fixed with 4% paraformaldehyde (Alfa Aesar, Haverhill, MA, USA), for 10 min, at room temperature. Cells were then permeabilized by incubation in a solution of PBS/Triton X-100 0.1% (v/v) for 10 min, blocked for 1 h with PBS/FBS 5% (v/v) and then incubated overnight, at 4 °C, in a humidified chamber, with the following primary antibodies: rabbit polyclonal anti-fibronectin antibody (1:300, Merck Life Science Srl, Milano, Italy, #F3648), mouse monoclonal anti-E-cadherin antibody (1:150, BD Transduction Laboratories, USA, #610,181) and mouse monoclonal anti αSMA antibody (1:300, Abcam, Cambridge, UK, #ab7817). Cells were then washed with PBS and incubated for 1 h with fluorophore-conjugated host-specific secondary antibodies: goat anti-mouse Alexa Fluor 488 and goat anti-rabbit Alexa Fluor 488 secondary antibodies (Thermo Fisher Scientific, Waltham, MA, USA). In addition, to label cells cytoskeleton, the secondary antibodies solution was supplemented with anti-Phalloidin-TRITC (1:1000, Merck Life Science Srl, Milano, Italy, #P1951). Afterwards, nuclei were stained by incubating cells with 1 μg/mL Hoechst 33342 dye (Thermo Fisher Scientific, Waltham, MA, USA) in PBS, 10 min at room temperature. Cell fluorescence was detected with the Zeiss AxioScop2 Fluorescent Microscope (Zeiss, Oberkochen, Germany) with a 40 × magnification objective. The ImageJ software (NIH, Bethesda, MD, USA), was used for the analysis of all the images acquired.

### Cu-dependent Superoxide dismutase (SOD1) activity

After treatments, cells were suspended in hypotonic PBS (1:2 dilution in water), lysed by sonication and centrifuged at 23000 × g, at 4 °C, for 30 min. After protein content determination by Lowry colorimetric assay (DC™ ProteinAssay, BioRad, Hercules, CA, USA), 50 µg of proteins were separated on 7.5% polyacrylamide PAGE under non-denaturating conditions. SOD1 activity was visualized by the inhibition of gel staining due to the conversion of nitroblue tetrazolium to formazan, as previously reported [25].

### Matrix metalloproteinases (MMPs) activity measurement by gelatin zymography

After treatments, cell medium was collected and centrifuged at 400 × g, at 4 °C, for 5 min to remove floating cells and debris. The supernatant was kept and mixed with 4 × sample buffer (250 mM Tris–HCl, pH 6.8, 40% glycerol; 8% SDS and 0.01% bromophenol blue). 40 μl of each sample were loaded onto 10% SDS–polyacrylamide gels containing 0.1% gelatin (Merck Life Science Srl, Milano, Italy). After electrophoresis, gels were incubated in the renaturing solution [2.5% Triton X-100 (Merck Life Science Srl, Milano, Italy l)], for 30 min, at room temperature, and then incubated in the developing buffer [50 mM Tris–HCl (pH 7.8), 200 mM NaCl, 5 mM CaCl_2_, 0.02% Triton X-100] overnight, at 37 °C. Gels were then stained with 0.5% Coomassie blue R250 (Merck Life Science Srl, Milano, Italy) for 1 h, and incubated in a destaining solution [10% methanol and 5% acetic acid (Merck Life Science Srl, Milano, Italy)]. The activity of MMPs was quantified by analyzing the intensity of colorless areas in the gels with the ImageJ software. Data are expressed as the mean from at least three independent experiments.

### Cell migration evaluation by wound healing and Boyden chambers assays

For assessing the cells migratory ability in a scratch assay, MDA-MB-231 and SK-BR-3 cells (0.3 × 10^4^ cell/cm^2^) were seeded in 24 well plates. After 72 h, the cell monolayer was scratched using a pipette tip through the central axis of the plate. Migration of the cells into the scratch was digitally documented after 24 and 48 h, and relative migratory activity was calculated measuring the cell-free areas. The wound closure areas were visualized under an inverted microscope with a 20 × magnification.

Cell migration was performed using Boyden chambers, with an 8.0 μm pore size (Corning, NY, USA). MDA-MB-231 and SK-BR-3 cells (0.5 × 10^4^ cell/well) were suspended in FBS-free media and loaded into the upper chamber, in the absence or presence of 125 µM TRIEN or 5 µM TTM. The lower chamber was filled with a complete medium supplemented with 20% FBS. After 24 h (for MDA-MB-231 cells) or 48 h (for SK-BR-3 cells) of incubation (37 °C; 5% CO_2_), cells adherent to the underside of the filters were fixed and permeabilized with 70% ethanol, washed with PBS, stained with 0.25% crystal violet (Merck Life Science, Srl, Milano, Italy). Cells in four random fields at magnification 20 × were counted.

### Real Time-PCR (RT-PCR) assay

Total RNA was extracted from treated cells using TRIzol Reagent (Invitrogen, Waltham, MA, USA) and 2.5 µg of RNA were reverse transcribed using Moloney murine leukemia virus (MMLV) reverse transcriptase (Promega, Madison, WI, USA). Afterwards, a RealTime PCR was carried out in 20 µL of final volume containing 10 ng of cDNA, 5 µM of each primer (Table 2) and 50% SYBR green (Kapa SYBR Fast qPCR kit; Kapa Biosystems, Roche, Wilmington, MA, USA) in a StepOnePlus Real-Time PCR System (Thermo-Fisher). The β-actin gene was used as an internal reference gene. Relative expression was evaluated using the 2^−ΔΔCt^ method. The primer sequences used in RT-qPCR analysis are listed in Table 2.

**Table 2:** RT-qPCR primers

Gene	Primers
*FN*	F: 5’-AGCCGAGGTTTTAACTGCGA-3’ R: 5’-CCCACTCGGTAAGTGTTCCC-3’
*SNAI1*	F: 5’-CCAGTGCCTCGACCACTATG-3’ R: 5-CTGCTGGAAGGTAAACTCTGG-3’
*SNAI2*	F: 5’-CCAAGCTTTCAGACCCCCAT-3’ R: 5’-GAAAAAGGCTTCTCCCCCGT-3’
*TWIST1*	F: 5’- GCTTGAGGGTCTGAATCTTGCT-3’ R: 5’- GTCCGCAGTCTTACGAGGAG-3’
*ZEB1*	F: 5’- CAGCTTGATACCTGTGAATGGG-3’ R: 5’- TATCTGTGGTCGTGTGGGACT-3’
*ACTB* [26]	F: 5’- ACCACCATGTACCCTGGCATT-3’ R: 5’- CCACACGGAGTACTTGCGCTCA-3’

### Metabolomic analysis

Deuterated reagents (methanol (CD_3_OD), chloroform (CDCl_3_)) and deuterium oxide (D_2_O) were purchased from Cambridge Isotope Laboratories, Inc.; 3-(trimethylsilyl)propionic-2,2,3,3-d4 acid sodium salt (TSP) was obtained from Merck & Co, Montreal, Canada. For the extraction of aqueous and organic metabolites [27] cell pellets and culture media were extracted according to protocol previously described [30]. Briefly, for samples preparation related to the intracellular metabolome, cell pellets were suspended in ice-cold extraction solvents [methanol/chloroform/water (1:1:1)] and vigorously vortexed. At least 24 h after, polar and lipid phases were separated by centrifugation at 20000 × g at 4 °C for 30 min. The polar methanol/water phase containing water soluble cellular metabolites was lyophilized by using a rotary evaporator (Savant RTV 4104 freeze dryer), while the organic phase (lipid phase) was collected in tube and chloroform was evaporated under nitrogen gas flow. Both phases of extracts obtained for each sample, treated and not, were stored at -20 °C. For preparation of the samples to estimate the extracellular metabolome, culture media extraction were performed by adding ice-cold extraction solvent [10 volumes of ethanolic solution (ethanol:water 77:23, v/v)] to each samples (0.5 mL) and stored at -20 °C for at least 24 h. Afterwards, the samples were centrifuged at 14000 × g for 30 min and the supernatant obtained was then freeze-dried in a Savant RTV 4104 freeze dryer.

The aqueous fraction from cells were reconstituted in 700 μL D_2_O using TSP (0.1 mM) as NMR internal standard whereas lipid fraction from cells was suspended in a CD_3_OD/CDCl_3_ solution (2:1 v/v) with 0.05%of tetramethylsilane (TMS) as internal reference. High-resolution ^1^H-NMR analyses were performed at 25 °C at 9.4 T and 14 T Bruker AVANCE spectrometers (Karlsruhe, Germany, Europe) on aqueous and organic cell extracts using acquisition pulses, water pre-saturation, data processing, and peak area deconvolution as previously described [27]. The absolute quantification of aqueous metabolites, determined by comparing the integral of each metabolite to the integral of reference standard TSP and corrected by respective proton numbers for metabolite and TSP, was expressed as nmoles/10^6^ cells and tissue and then converted into metabolite percentage (relative to total metabolites evaluated in each sample). Relative quantification of lipid signals (integral) in organic fractions was normalized to the number of cells.

### Bioinformatic analyses

The differences in RAC-alpha serine/threonine-protein kinase (i.e., AKT; UniProt ID P31749) and Copper Chaperone for Superoxide dismutase 1 (i.e., CCS; UniProt ID O14618) expression patterns between distinct molecular subtypes of Breast Cancer (Basal, HER2, Luminal, Luminal A, Luminal B, and TNBC) was investigated, analyzing data from the Gene Expression database of Normal and Tumor tissues 2 (i.e., GENT2). (http://gent2.appex.kr/gent2/; accessed on 24 January 2022) [28] In addition, the data for mRNA expression and the “Neoplasm Histologic Grade” were retrieved from cBioPortal by selecting all studies on Breast Cancer available (https://www.cbioportal.org/; accessed on 24 January 2022) [29, 30]. All data were statistically elaborated by using One-Way ANOVA and statistical significance was set at *p* < 0.0001.

The correlation between breast cancer patients’ survival and *AKT* and *CCS* mRNA was performed using three databases. First, we looked at the effect on the Overall Survival (OS) using the GENT2 database (http://gent2.appex.kr/gent2/; accessed on 24 January 2022) [28]. Then, we evaluated the Progression-Free Interval (PFI) of phosphorylated AKT protein (*i.e.*, AKT pS473) in Breast Cancer through data from the Cancer Proteome Atlas portal (i.e., TCPA portal) (https://www.tcpaportal.org/; accessed on 23 January 2022) [31, 32]. Finally, the Kaplan–Meier plotter was used to examine the influence of CCS expression in Breast Cancer on Relapse-Free Survival (RFS) (https://kmplot.com/analysis/; accessed on 23 January 2022) [33].

### Statistical analysis

Results were reported as mean value ± standard error of the mean (SEM) of measurements obtained by independent experiments (n ≥ 3). Statistical significance was evaluated by Student’s t test, one-way ANOVA or two-way ANOVA followed by post hoc Sidak’s or Tukey’s multiple comparisons test, using the GraphPad Prism software (GraphPad Software, San Diego, CA, USA); a *p*-value < 0.05 was considered significant (*p < 0.05; **p < 0.01; ***p < 0.001; ****p < 0.0001).

## Results

### Analysis of proteins involved in Cu homeostasis in cell lines of breast cancer and their modulation by treatment with the Cu-chelator TRIEN

**Fig. 1:**
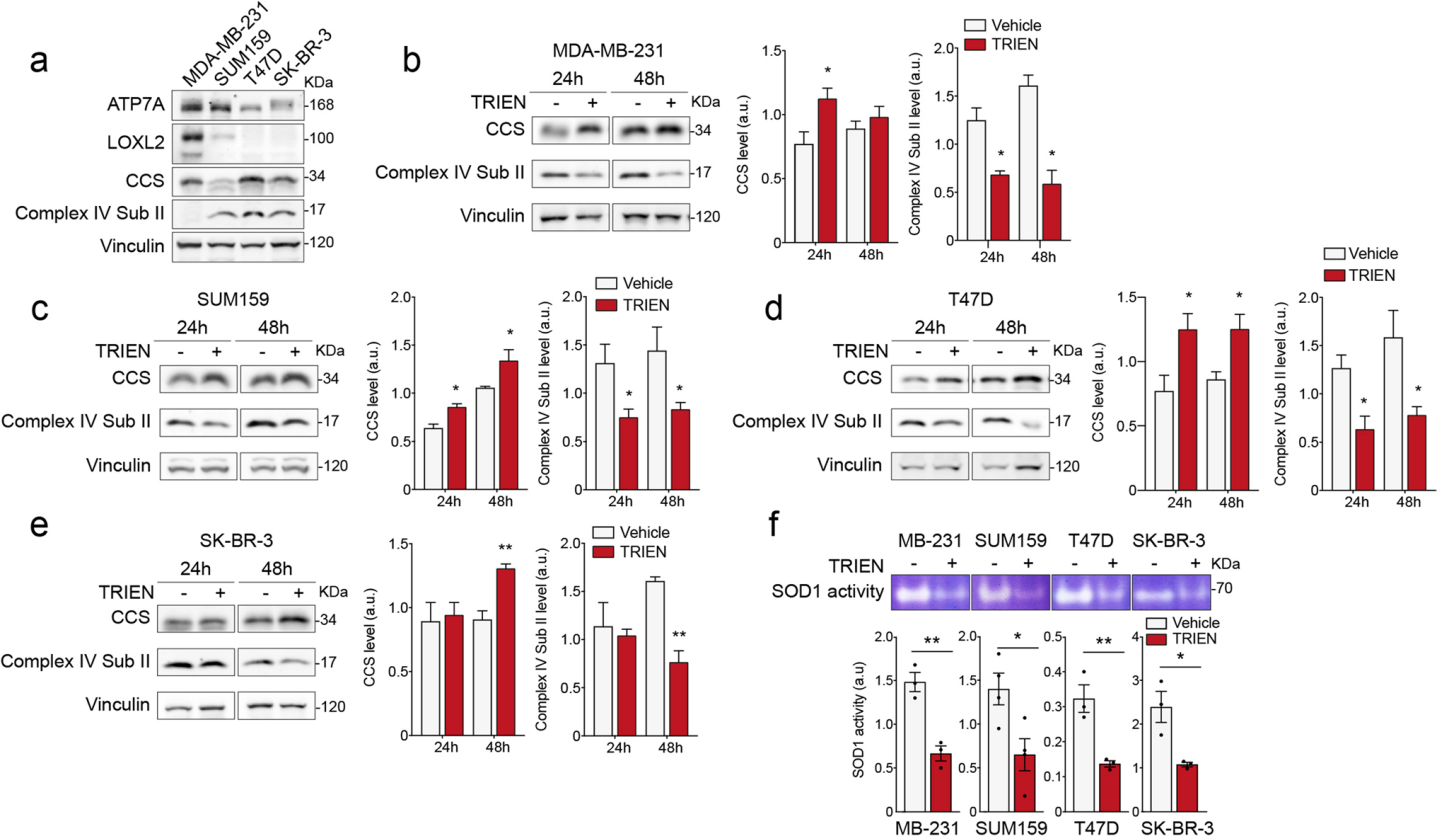
Basal levels of cuproproteins in different breast cancer cell lines and their modulation following TRIEN treatment. Cell extracts obtained from MDA-MB-231, SUM159, T47D and SK-BR-3 were applied to SDS–PAGE followed by Western blot with monoclonal antibodies for the detection of basal levels of **(a)** ATP7A, LOXL2, CCS and of the subunit II of cytochrome c oxidase. The level of CCS and the of the subunit II of cytochrome c oxidase, was measured by Western blot (**left panels;** correspondent densitometric analysis in the **right panels**), following 24 or 48 h of 125 µM TRIEN treatment in **(b)** MDA-MB-231, **(c)** SUM159, **(d)** T47D, and **(e)** SK-BR-3 cells. Twenty micrograms of proteins were loaded on each lane. Vinculin was used as loading control. One representative blot is shown for each antigen out of at least three independent experiments. **(f, upper panel).** SOD1 activity was assessed in the different cell lines lysates using an in-gel assay, under non-denaturing, SDS-free conditions, after 48 h of treatment with 125 µM TRIEN; fifty micrograms of proteins were applied to each lane. One representative SOD1 activity assay is shown out of at least three different experiments; **(f, lower panel)** densitometric analysis of the activity assay. Data are presented as a mean ± SEM. Student's t-test *p < 0.05, **p < 0.01 with respect to the untreated cells only

We started by characterizing the basal level of Cu-dependent proteins which are reported to play a role in cancer development and spreading (*i.e.*, ATP7A, LOXL2, CCS and Cytochrome c Oxidase) in the cells under study [34] (Fig. 1a). Among them, ATP7A levels were higher in both TNBC cell lines (*i.e.,* MDA-MB-231 and SUM159), whereas intracellular LOXL2 was abundant only in MDA-MB-231 (Fig. 1a). The abundance of the Subunit II of Cytochrome c Oxidase (the Complex IV of the mitochondrial respiratory chain) was much lower in MDA-MB-231 than in the other cell lines (Fig. 1a), while the level of CCS, the Copper Chaperone for SOD1, was lower in SUM159 cells than in the other cell lines.

To achieve reduced bioavailability of Cu in our experimental cell model in order to observe the effects on EMT markers, we treated the cells with a well-established Cu-chelator, triethylenetetramine (TRIEN). To check possible effects of TRIEN on cell viability, first of all we performed an MTS assay. We found that TRIEN did not affect cell viability when added to the cell medium at concentrations ranging from 2 to 1000 μM for 72 h (see supplementary Figure S1). On the basis of these results and referring to previously published work from our laboratory [25, 35], we chose 125 μM TRIEN concentration for the next experiments.

The effect of TRIEN treatment on Cu bioavailability was assessed by analyzing Cu-binding proteins considered as sensors of intracellular Cu levels (Fig. 1b-f). We measured the protein level of CCS, which is known to increase under condition of Cu depletion [36], the level of the Subunit II of cytochrome c oxidase, which undergoes degradation upon Cu shortness [25], and the activity of SOD1 [25].

In MDA-MB-231, SUM159 and T47D cells, 125 μM TRIEN treatment for 24 h or 48 h caused increase of CCS protein paralleled by the reduction of the Subunit II of the Complex IV (Fig. 1b, c, and d). However, in the HER2-overexpressing cell line (SK-BR-3) the same effects on those proteins were observed only at 48 h of TRIEN treatment, suggesting a slower response of these cell line (Fig. 1e). To further validate the effectiveness of TRIEN in reducing Cu bioavailability, we also measured the activity of SOD1, which utilizes Cu as a cofactor, upon 48 h TRIEN addition. Indeed, TRIEN treatment significantly impaired SOD1 activity in all the cell lines examined (Fig. 1f) [25, 37]. Taken together, these results confirmed that TRIEN efficiently reduced Cu bioavailability, but that such effect requires different times, depending on the cell model.

Thus, in the subsequent experiments, we treated with TRIEN MDA-MB-231, SUM159 and T47D cells for 24 h and the SK-BR-3 cells for up to 48 h.

### Cu bioavailability affects the epithelial/mesenchymal markers

**Fig. 2:**
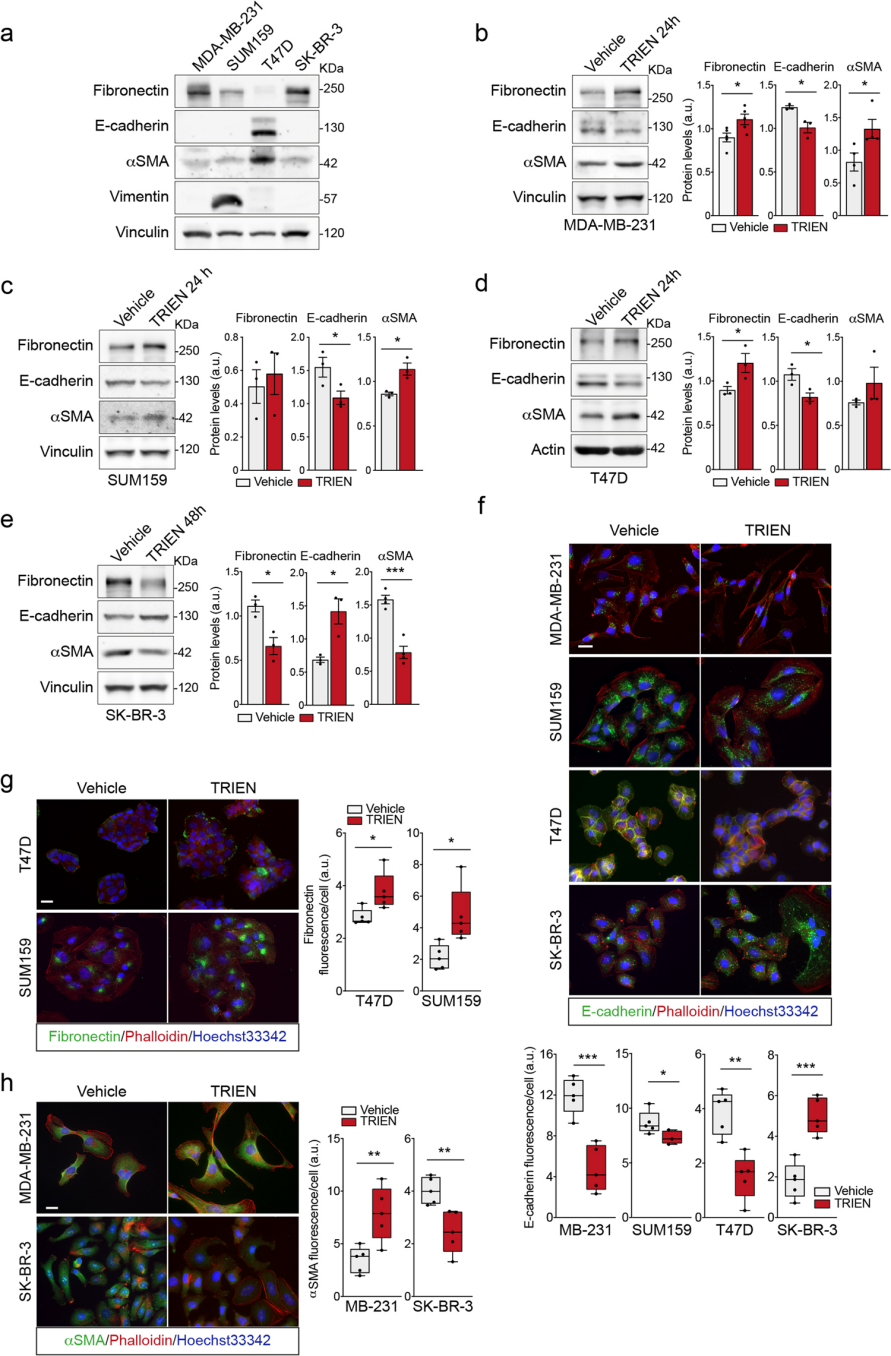
Cu bioavailability modulates the level of the epithelial to mesenchymal transition hallmarks. The basal levels of fibronectin, E-cadherin, αSMA and vimentin were measured in cell extracts from (**a**) MDA-MB-231, SUM159, T47D and following 24 h exposure to 125 µM TRIEN for (**b**) MDA-MB-231, (**c**) SUM159, (**d**) T47D and 48 h after TRIEN treatment for (**e**) SK-BR-3. Twenty micrograms of proteins were loaded on each lane. Vinculin or actin were used as loading controls. (**f**) Immunofluorescence detection of E-cadherin in MDA-MB-231, SUM159, T47D 24 h after TRIEN exposure and SK-BR-3 cells 48 h after treatment with 125 µM TRIEN (upper panels) and its corresponding signal analysis (lower panels). (**g**) Fibronectin immunofluorescence of T47D and SUM159 **(left panels)** and its corresponding signal analysis **(right panels)**. (**h**) αSMA immunofluorescence in MDA-MB-231 and SK-BR-3 **(left panels)** and its corresponding signal analysis **(right panels)**. Phalloidin was used to label F-actin whilst nuclei were labeled with DAPI. Calibration bars corresponds to 100 μm in the immunofluorescence images; 40 × magnification. One representative blot/image and the corresponding signal analysis is shown for each antigen, out of at least three independent experiments. Student's t-test *p < 0.05, **p < 0.01 and ***p < 0.001 with respect to the untreated cells only. Data are presented as a mean ± SEM

The EMT profile of each breast cancer cell line under study was evaluated by measuring the protein levels of the epithelial marker E-cadherin and of the mesenchymal markers fibronectin, vimentin and αSMA by Western Blot analysis (Fig. 2a). We found that MDA-MB-231, SUM159 and SK-BR-3 are mainly mesenchymal, expressing higher levels of mesenchymal markers. MDA-MB-231 cells express high levels of fibronectin and are also characterized by the presence of αSMA; SUM159 cells are endowed by high level of vimentin and also by the expression of fibronectin and αSMA and SK-BR-3 are characterized by the expression of fibronectin and, to a lower extent, of αSMA (Fig. 2a). On the contrary, T47D cells have the highest level of E-cadherin as well as of αSMA, thus showing a mixed epithelial-mesenchymal phenotype (Fig. 2a). Indeed, when sensitivity of Western blotting was improved (Fig. 2b, c and e), E-cadherin could be detected also in MDA-MB-231, SUM159 and SK-BR-3, although to a much lesser extent than T47D.

Considering the putative importance of Cu imbalance in tumor [2, 34, 38], we hypothesized that it could also be involved in the EMT process. Hence, we investigated the capacity of TRIEN in modulating the EMT phenotype in our selection of breast cancer cell lines, by analyzing the changes of the epithelial/mesenchymal markers (E-cadherin, fibronectin, vimentin and αSMA) specifically expressed in each cell line, by Western blot and immunofluorescence analyses (Fig. 2b-h). Interestingly, we observed that TRIEN had an opposite effect, depending on the specific cell line. Indeed, in MDA-MB-231, SUM159 and T47D cells 24 h TRIEN treatment induced a decrease of the epithelial marker E-cadherin (Fig. 2b-d) and a rise of mesenchymal markers such as fibronectin and αSMA (Fig. 2b-d), thus directing the cells towards a mesenchymal phenotype. By contrast (Fig. 2e), SK-BR-3 cells treated with 125 μM TRIEN for 48 h showed a significant increase of the epithelial marker E-cadherin and a drastic reduction of mesenchymal markers fibronectin and αSMA, thus reinforcing the concept that this cell line responds more slowly to TRIEN treatment and, most of all, acquires completely different characteristics with respect to the other cell lines. Immunofluorescence analysis confirmed the differential regulation of E-cadherin (Fig. 2f), fibronectin (Fig. 2g) and αSMA levels (Fig. 2h), following Cu depletion, in the different cell lines.

To further validate that Cu modulates the EMT profile of breast cancer cells, to restore Cu bioavailability, MDA-MB-231 and SK-BR-3 cells, after TRIEN treatment for 24 and 48 h, respectively, medium with TRIEN was removed and cells incubated in a medium supplemented with 100 μM CuSO_4_, for up to 3 h (Figure S2). Of note, we observed a complete reversal of the TRIEN-induced effects previously described in both cell lines. In MDA-MB-231 we observed a decrease to basal levels of fibronectin, αSMA, and CCS and an increase of E-cadherin (Figure S2a), while in SK-BR-3 cells the treatment with CuSO_4_ induced an increase in fibronectin, αSMA and subunit II of complex IV and a decrease in E-cadherin levels (Figure S2b). In summary, by restoring the bioavailability of Cu, both MDA-MB-231 and SK-BR-3 cells revert to their original phenotype.

### Treatment with TRIEN alters the migration tendency of breast cancer cells

**Fig. 3:**
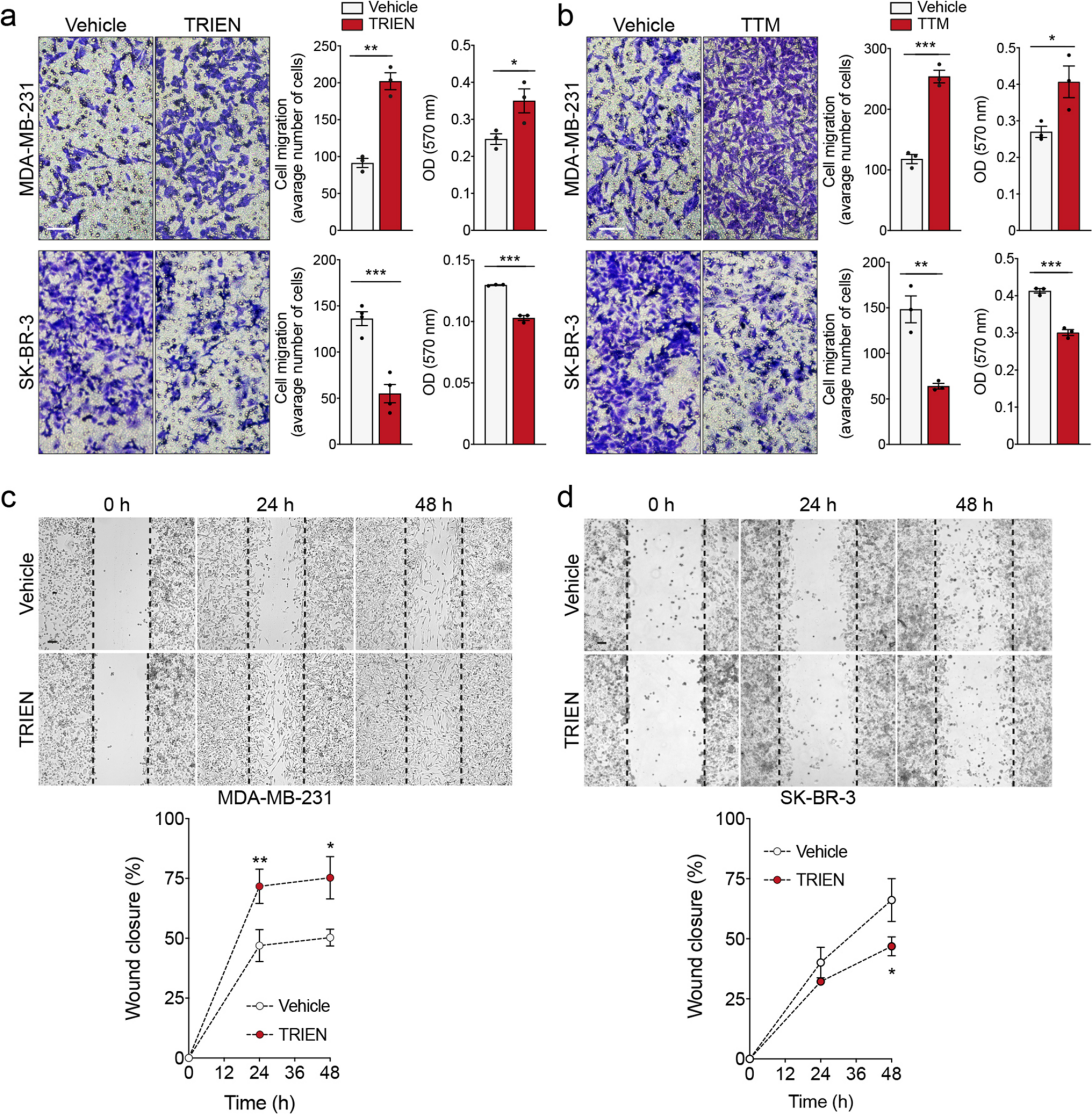
Decreased Cu bioavailability differently affects the migration of MDA-MB-231 and SK-BR-3 breast cancer cell lines. (**a** **and** **b**, **upper panel**) MDA-MB-231 and **(a and b, lower panel)** SK-BR-3 cells were assayed for in vitro migration using a Boyden chamber. After 24 h of exposure to (**a**) 125 µM TRIEN and (**b**) 5 µM TTM, migrated cells per field were stained with crystal violet and counted. One representative phase contrast image (20 × magnification) is shown, out of at least three independent experiments. Student's t-test *p < 0.05, **p <0.01, ***p < 0.001 with respect to the untreated cells only. Data are presented as a mean ± SEM. Wound-healing assay in (**c**) MDA-MB-231 and (**d**) SK-BR-3 cells treated with TRIEN for up to 48 h. A single scratch was made in the center of the cell monolayer and the wound closure areas visualized under an inverted microscope with 20 × magnification **(upper panel)**. Cell motility was quantified by measuring the distance between the invading front of cells in at least 5 random selected microscopic fields for each single condition and time point **(lower panel)**. Data are presented as a mean ± SEM (*n* ≥ 3, ***p < 0.001, Two-Way ANOVA). Calibration bars 100 μm in transwell migration **(a and b)** and wound healing assay **(c and d)**

To investigate whether Cu depletion could alter the migratory ability of breast cancer cells, we performed both the wound healing and the transwell migration assays. As expected, 24 h TRIEN treatment enhanced the migration of MDA-MB-231 with respect to untreated cells (Fig. 3a, upper panel, and Fig. 3c), demonstrating that in these cells, the acquisition of mesenchymal markers is associated with an increased cell motility. Conversely, in SK-BR-3 cells 48 h TRIEN treatment reduced cell migration trend (Fig. 3a, lower panel, and Fig. 3d), furtherly confirming the peculiar behavior of this cell line shown in Fig. 2.

To confirm that the effects of TRIEN on cell migration were linked to the reduction of Cu bioavailability, we repeated the transwell migration test using 5 μM tetratiomolybdate (TTM), another well-established Cu-chelator [39], in both cell lines. The concentration of TTM was selected based on the literature [40, 41]. TTM promoted cell migration in MDA-MB-231 (Fig. 3b, top panel) and reduced cell motility of SK-BR-3 cells (Fig. 3b, lower panel), effects which are superimposable on those produced by TRIEN.

### Cu chelation modulates the activation of kinases involved in EMT and the transcription factor controlling EMT markers expression

**Fig. 4:**
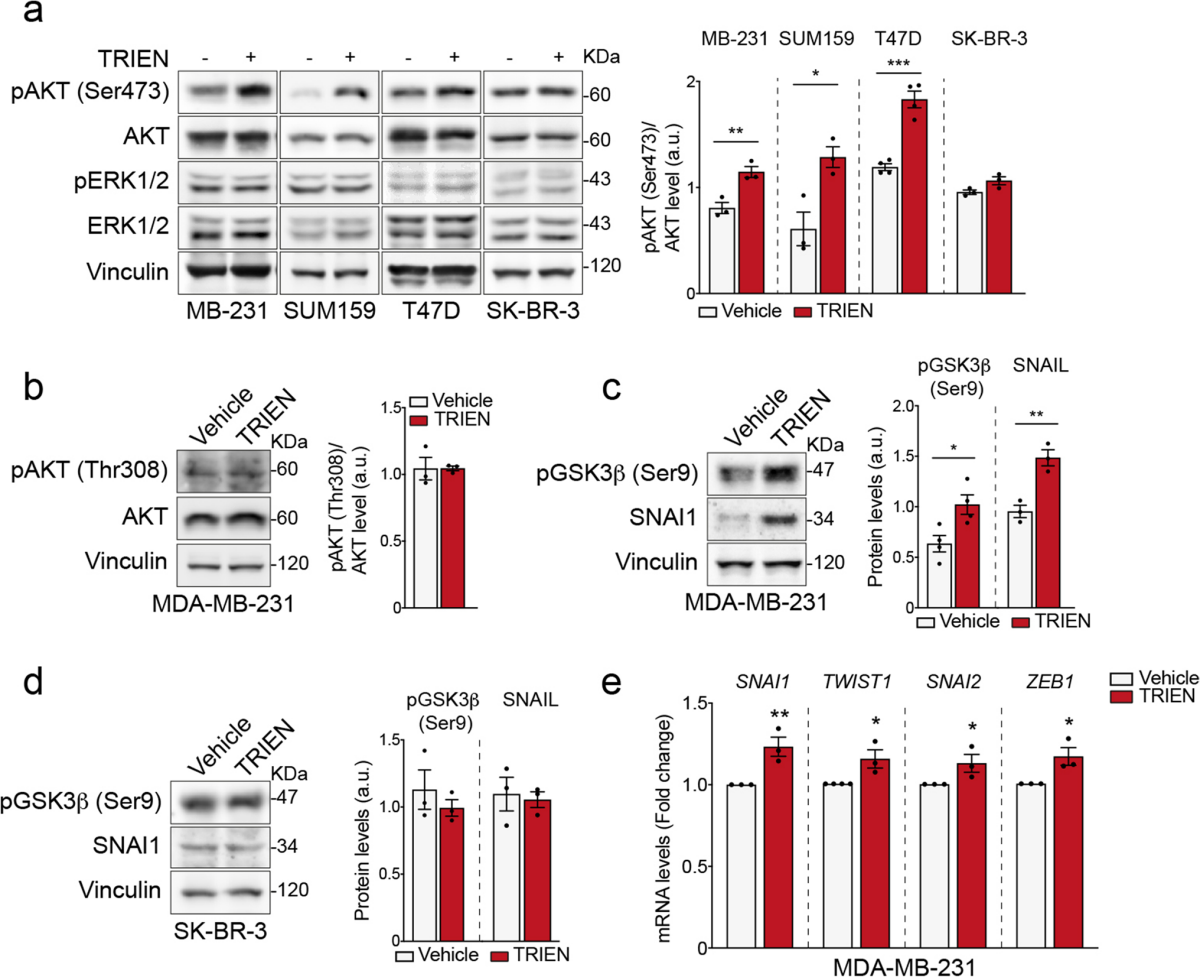
Cu chelation modulates the activation of the AKT/GSK3β/SNAIL axis. **(a, left panel**) Western Blot of phosphorylated AKT at Ser473 (pAKT Ser473), total AKT (AKT), phosphorylated ERK1/2 (pERK1/2) and total ERK 1/2 (ERK1/2) in MDA-MB-231, SUM159, T47D and SK-BR-3 following 24 h exposure to 125 µM TRIEN for MDA-MB-231, SUM159, T47D and 48 h after TRIEN treatment for SK-BR-3. **(a, right panel)** densitometric analysis of phosphorylated AKT at Ser473 residue (pAKTSer473)/total AKT. **(b, left panel)** Western blot and **(b, right panel)** densitometric analysis of pAKT at Thr308 (pAKT Thr308) and total AKT (AKT) in MDA-MB-231; **(c, left panel)** Western blot and **(c, right panel)** densitometric analysis of phosphorylated GSK3β at Ser9 (pGSK3β Ser9) and SNAI1 in MDA-MB-231and **(d)** SK-BR-3 cells. Twenty micrograms of proteins were loaded on each lane. Vinculin was used as loading control. One representative blot is shown for each antigen, data are presented as a mean ± SEM (*n* ≥ 3, Student's t-test *p < 0.05; **p < 0.01 ***p < 0.001). **(e)** The level of the EMT-TFs SNAI1, TWIST1, SNAI2 and ZEB1 transcripts in MDA-MB-231 cells were evaluated by qPCR analysis following 24 h exposure to 125 µM TRIEN. Results were expressed with respect to the control (vehicle), defined as 1. Data were reported as mean ± SEM of three independent measurements (*p < 0.05, **p < 0.01 vs vehicle)

It has been reported that Cu binds, allosterically modulates and activates MEK1, which in turn triggers the Extracellular signal-Regulated Kinase 1/2 (ERK1/2) [12]. Thus, we first evaluated the activation of ERK1/2 through Western blot analysis. Interestingly, as shown in Fig. 4a, 125 μM TRIEN (24 h for MDA-MB-231, SUM159 and T47D and 48 h for SK-BR3) did not alter ERK 1/2 phosphorylation status in all breast cancer cell lines. Furthermore, we investigated the modulation by Cu of the kinase axis AKT/GSK3β, known to be involved in the induction of EMT [7]. Intriguingly, AKT was phosphorylated at Ser473 in MDA-MB-231, SUM159 and T47D (Fig. 4a). SK-BR-3 cells behaved differently, since AKT was not phosphorylated upon TRIEN treatment (Fig. 4a). To further investigate the activation mechanism of AKT in MDA-MB-231 we analyzed AKT phosphorylation at Thr308 (Fig. 4b), demonstrating lack of involvement of Thr308 in Cu-modulated AKT activity.

Thus, in MDA-MB-231 cells we investigated the AKT signaling cascade, by evaluating the phosphorylation of its downstream target, GSK3β. It is well known that AKT phosphorylates GSK3β in its Ser9 suppressing its activity [42]. We found that AKT phosphorylation resulted in an increased phosphorylation of GSK3β in its Ser9 and consequently, leads to an up-regulation of SNAI1, which undergoes degradation when phosphorylated by active GSK3β [43] both in terms of protein level (Fig. 4b) and of mRNA (as shown below, Fig. 4d).This confirms that, upon TRIEN treatment, AKT activation and GSK3β inactivation occur in MDA-MB-231 cells. By contrast, in SK-BR-3 cells, the phosphorylation status of GSK3β remained unchanged as well as SNAI1 protein levels (Fig. 4c).

The mechanism underlying the modulation of the investigated EMT markers was studied in MDA-MB-231 by analyzing by RT-PCR the mRNA levels of the transcription factors (TFs) involved in EMT: SNAI1, SNAI2, TWIST1 and ZEB1. Indeed, we found that all these TFs were slightly upregulated following 24 h TRIEN exposure, with the strongest effect on SNAI1 (Fig. 4d).

### Protracted TRIEN treatment alters cell morphology and counteracts TGFβ-induced EMT

**Fig. 5:**
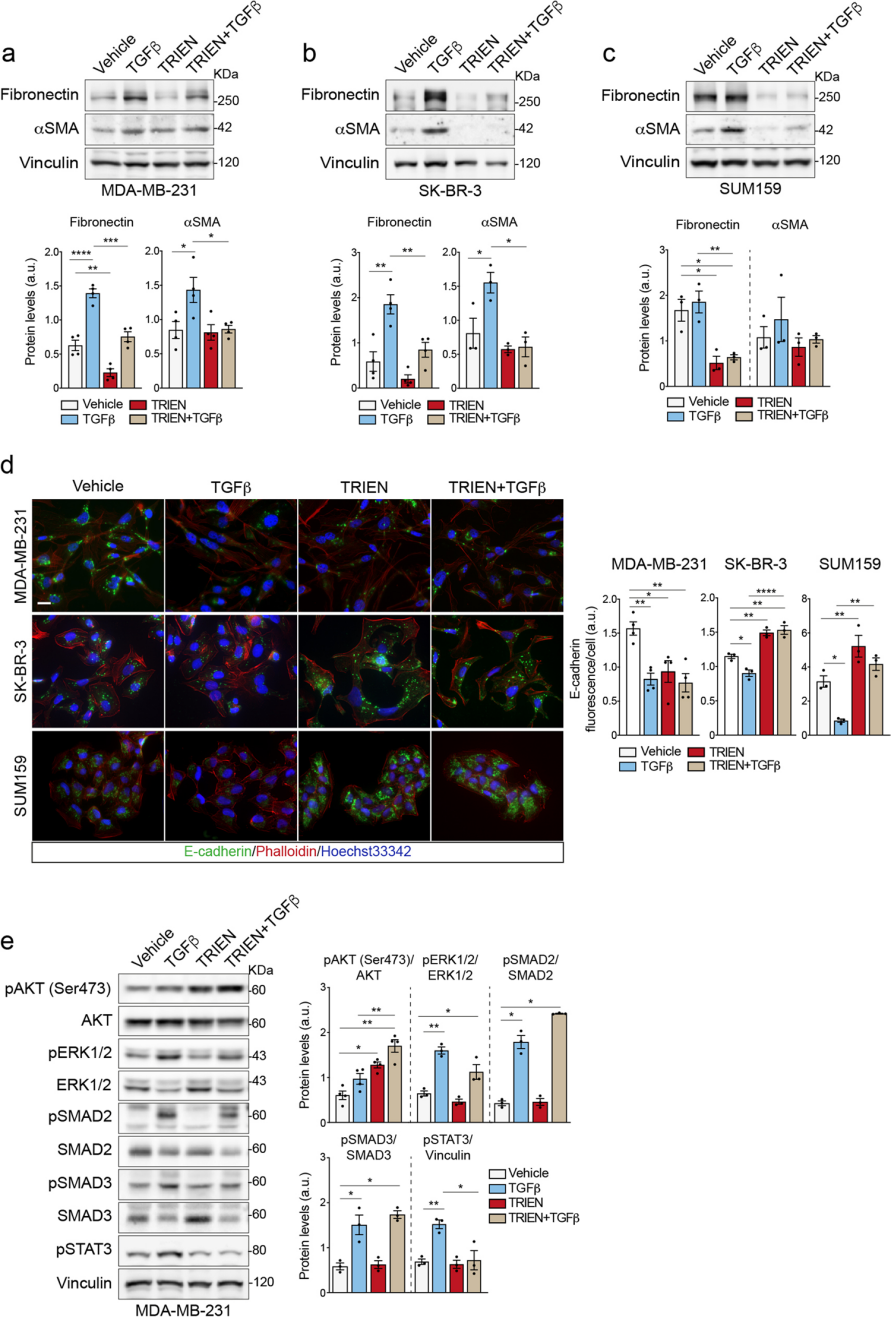
Effects of prolonged TRIEN exposure and TGFβ treatment on EMT markers and breast cancer cells phenotype. Western blot **(upper panels)** and densitometric analysis **(lower panels)** of fibronectin and αSMA in **(a)** MDA-MB-231, **(b)** SK-BR-3 and **(c)** SUM159 following 10 ng/ml TGFβ (24 h) and/or 125 µM TRIEN (6 days in total) treatment. 20 µg of proteins were applied to each lane. Vinculin was used as loading control. **(d)** Immunofluorescence images of E-cadherin in MDA-MB-231, SK-BR-3 and SUM159 cells **(d, left panels)** and their corresponding signal analysis **(d, right panels)**. Calibration bar: 100 μm. 40 × magnification. **(e)** Western blot **(e, left panel**) and densitometric analysis **(e**, **right panels)** of phosphorylated AKT at Ser473 (pAKT), total AKT (AKT), phosphorylated ERK1/2 (pERK1/2) and total ERK 1/2 (ERK1/2), phosphorylated SMAD2 (pSMAD2), total SMAD2 (SMAD2), phosphorylated SMAD3 (pSMAD3), total SMAD3 (SMAD3) and phosphorylated STAT3 (pSTAT3) in MDA-MB-231. Vinculin was used as loading control. One representative blot/image is shown for each antigen, data are presented as a mean ± SEM (*n* ≥ 3, One-way ANOVA, *p < 0.05; **p < 0.01 ***p < 0.001, ****p < 0.0001)

Next, we investigated the effect of 24 h of treatment with TGFβ, the main inducer of EMT [44], either alone or by adding it to cells already treated with TRIEN for 5 days to achieve Cu depletion (*i.e.*, treatment with TRIEN was prolonged up to 6 days in total). At first, to dissect the effects of prolonged TRIEN treatment (6 days) and to assess a possible difference with the short-time treatments (24/48 h) (see data shown in Fig. 2) on the EMT phenotype, we measured the levels of fibronectin and of αSMA (by Western blot). Interestingly, the exposure of MDA-MB-231 (Fig. 5a), SK-BR-3 (Fig. 5b) and SUM159 (Fig. 5c) cells to 6 days TRIEN produced a decrease of fibronectin whereas the levels of αSMA were comparable to untreated cells. A possible mechanism involved in the decrease of cellular fibronectin could be its excretion in the extracellular medium. However, the measurement of fibronectin in the MDA-MB-231 and SUM159 cell media also showed a decrease with respect to untreated cells (Figure S3a, b). Thus, both the intracellular and the extracellular forms of fibronectin showed a drastic decrease following 6 days of treatment with TRIEN. Furthermore, in this experimental contest, the epithelial marker E-cadherin, measured by immunofluorescence, decreased in MDA-MB-231 cells (Fig. 5d, upper panel) whereas it increased in SK-BR-3 and SUM159 cells, (Fig. 5d, middle and lower panel, respectively). In supplementary Figure S4a, a representative Western blot analysis of E-cadherin expression levels in MDA-MB-231 shows that the results were superimposable on those obtained by immunofluorescence. As a further confirm, in supplementary Figure S4b, fibronectin distribution observed by immunofluorescence in MDA-MB-231 cells is shown. Also in this case the results were superimposable on those obtained by the Western blot assay.

Then, we analyzed the effect of 24 h TGFβ treatment alone (10 ng/ml), in Cu-adequate cells. As expected, TGFβ promoted the acquisition of a mesenchymal phenotype in MDA-MB-231, SUM159 and SK-BR-3 cells, upregulating fibronectin and αSMA and downregulating E-cadherin (see Western blot and immunofluorescence analyses shown in Fig. 5a-d). Conversely, in all Cu-depleted cell lines for up to 6 days, TRIEN counteracted the effect produced by TGFβ (Fig. 5a-d).

In addition, upon TGFβ stimulation, in MDA-MB-231 and SUM159 the levels of fibronectin secreted in the cell media, were higher than in untreated cells (Figure S3 a, b) However, prolonged TRIEN treatment abolished TGFβ-induced fibronectin release (Fig. S3a,b).

Next, we investigated the occurrence of distinctive cellular morphological changes of the mesenchymal phenotype during treatment with TGFβ (alone for 24 h or after 6 days of TRIEN treatment). Indeed, growth factors push epithelial cells to acquire a spindle-shaped morphology with numerous filopodia [4]. In fact, after treatment with TGFβ, the MDA-MB-231 cells acquired a spindle shape, while in the same cells pretreated with TRIEN, TGFβ was no longer able to induce this morphological change (Figure S3c).

### Long-term Cu chelation influences TGFβ activation of non-canonical EMT mediators STAT3 and AKT in MDA-MB-231 cells

The activation of EMT, triggered by TGFβ, may proceed through “canonical” or “non-canonical” pathways, involving SMAD protein members or ERK1/2, AKT and STAT3, respectively [7]. To investigate which route was preferentially active under our experimental conditions, we measured these markers by Western blot analyses (Fig. 5e). As expected, TGFβ alone increased phosphorylation and activation of SMAD2/3, ERK1/2 and STAT3. On the contrary, no significant activation of AKT was revealed. However, when the cells were exposed for up to 6 days to TRIEN, alone or in combination with TGFβ, AKT was activated but not STAT3 (Fig. 5e). In synthesis, protracted TRIEN treatment affects the pathway involving AKT, even in the presence of TGFβ, but counteracts TGFβ-mediated activation of STAT3.

### Cu bioavailability and TGFβ influence ECM remodeling by impairing the activity of MMPs and LOXL2

**Fig. 6:**
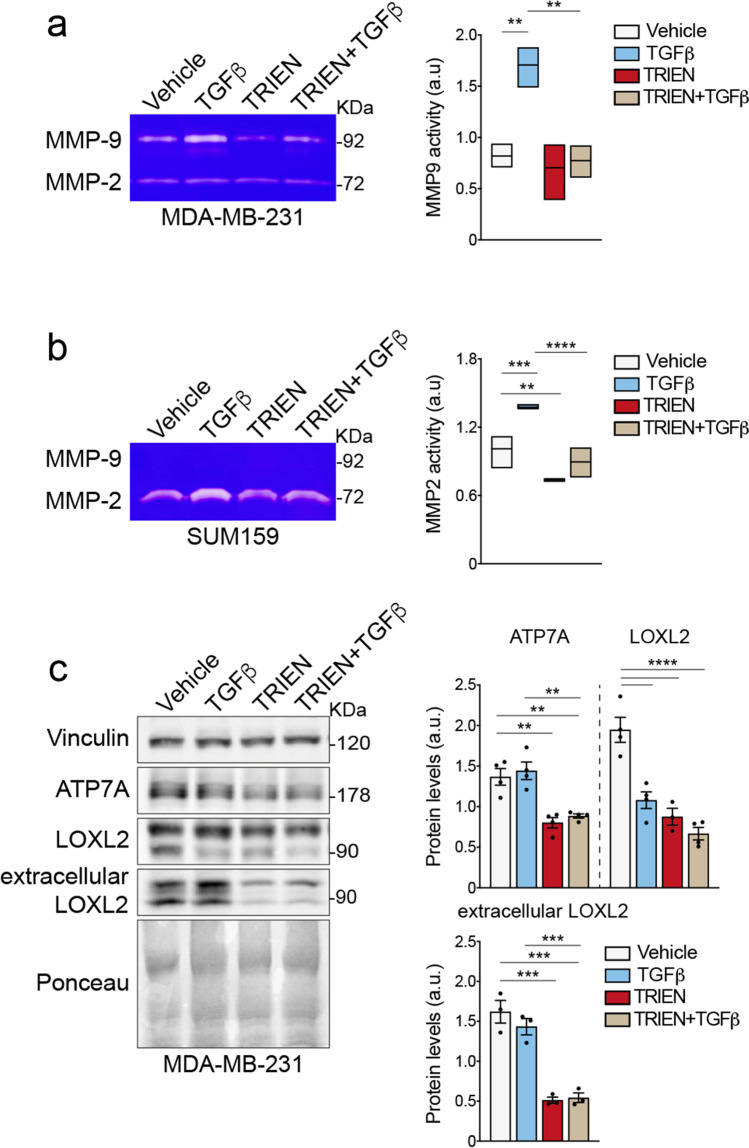
Prolonged TRIEN exposure impairs the activities of MMPs and the level of LOXL2, even in the presence of TGFβ**. (a, left panel)** Gelatin zymography assay performed in the cell media of MDA-MB-231 and** (b, left panel)** SUM159 showing the activity of MMP2 and MMP9 upon treatment with TGFβ (24 h) or 125 µM TRIEN (6 days), alone and in combination, and their densitometric analyses **(right panels)**. 40 μl of media was applied to each line. **(c, left panel)** Western blot and **(c, right panels)** densitometric analysis of ATP7A, LOXL2, and extracellular LOXL2 in MDA-MB-231, following 10 ng/ml TGFβ (24 h) or 125 µM TRIEN (6 days) treatment, alone and in combination. 40 µg of proteins or 25 µl were loaded on each lane. Vinculin was used as loading control; Ponceau S staining was used as loading control for the extracellular LOXL2. One representative image/blot is shown for each gelatin zymography assay/antigen, data are presented as a mean ± SEM (n ≥ 3, One-way ANOVA, **p<0.01, ***p<0.001, ****p<0.0001

Among the proteins involved in EMT, MMPs are required for ECM remodeling favoring metastasis formation [45]. Thus, we investigated the effect of prolonged TRIEN treatment and TGFβ-triggered EMT on the activity of MMP2/9 secreted in the cell media by a gelatin zymography assay. We observed that TGFβ treatment alone enhanced MMP9 activity in MDA-MB-231 cells and MMP2 activity in SUM159 cells (Fig. 6a,b). Protracted TRIEN treatment, alone or in combination with TGFβ, did not change MMPs activities (Fig. 6a,b). In SK-BR-3 cells, the activity of MMPs was not detectable, due to poor expression of the MMPs (data not shown).

Besides MMPs, also Cu-dependent enzymes belonging to the LOX family (LOXL1-4) are involved in the remodeling the ECM. It is also suggested that the P-type ATPase ATP7A promotes tumorigenesis and metastasis, since it supplies Cu to LOX [12]. Therefore, we tested whether the protracted reduction of Cu bioavailability in MDA-MB-231 could alter the levels of these pro-metastatic proteins. Notably, we observed the reduction of ATP7A and LOXL2 protein levels upon TRIEN treatment for 6 days (Fig. 6c), alone and in combination with TGFβ. On the contrary, following TGFβ treatment alone, ATP7A levels were unchanged whilst LOXL2 levels decreased. As for the activity of MMP2/9, we evaluated the level of LOXL2 in the cell medium. We found that TGFβ treatment did not affect secreted-LOXL2 levels, whilst protracted TRIEN treatment produced a significant reduction of secreted-LOXL2, even when cells were treated together with TGFβ.

### TRIEN treatment strongly affects TNBC MDA-MB-231 cells metabolism

**Fig. 7:**
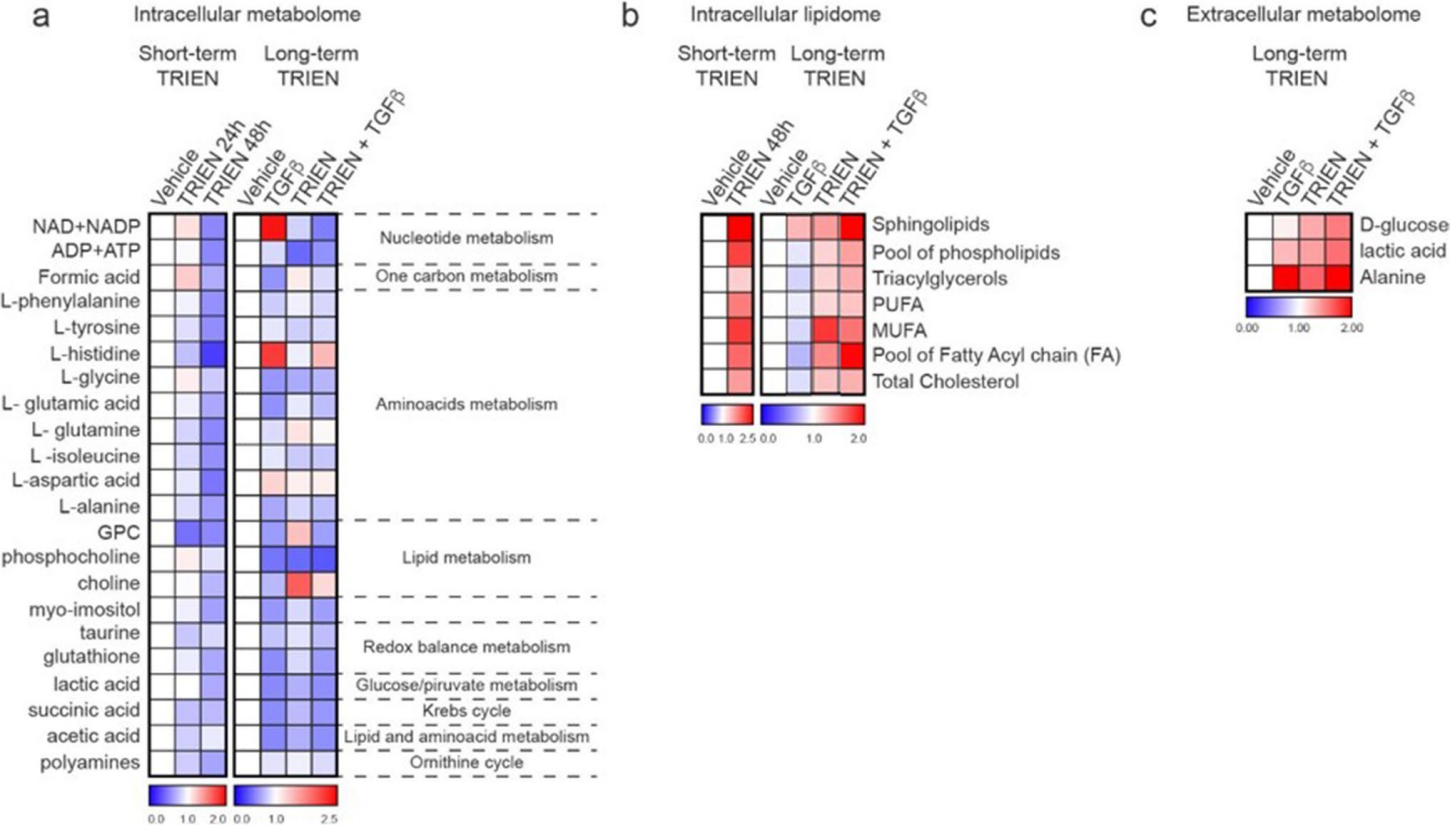
Effects of TRIEN and TGFβ treatment on the metabolic pathways of MDA-MB-231 cells. (**a**) NMR analysis of the intracellular aqueous metabolites and (**b**) intracellular lipidome in MDA-MB-231 following up to 48 h 125 µM TRIEN treatment **(left panel)** and following cells treatment for 24 h with 10 ng/ml TGFβ, alone or added to TRIEN-pretreated TNBC cells **(right panel)**. (**c**) NMR analysis of the extracellular metabolome of MDA-MB-231 cells correlated to the glucose/pyruvate metabolism, following cells treatment with 10 ng/ml TGFβ for 24 h, alone or added to 125 µM TRIEN-pretreated cells. Metabolomic data are presented as heat maps of fold change, the metabolites level of the untreated control is referred as 1

To understand whether TRIEN treatment translated into metabolic changes in the MDA-MB-231 cells, we analyzed their intracellular metabolism, by using NMR-metabolomics approach. We found that short- term TRIEN exposure induced biochemical changes in the metabolites fueling Krebs Cycle, such as aromatic amino acids, glutamic acid and aspartic acid (Fig. 7a, left panel). Indeed, a strong reduction of the energetic metabolism of the TNBC cells (ATP + ADP and NAD + NADP) was only observed after 48 h exposure to TRIEN. Interestingly, we found an increase in the level of formic acid (Fig. 7a, left panel), which is involved in the synthesis of nucleotides. Furthermore, Cu depletion induced an increase of neutral lipids (cholesterol, fatty acids and degree of unsaturation) as well as an accumulation of the polar lipids, such as phospholipids and sphingolipids (Fig. 7b, left panel, and Table 3), likely due to an impairment of mitochondria activities, such as lipid β-oxidation.

**Table 3:** NMR: Relative quantification (Integral/1 × 10^6^ cells) of lipid metabolites involved in the main pathways detectable by NMR spectroscopy (14 T), in organic fraction, of MDA-MB-231 cells (*n* = 2), following 48-h treatment with TRIEN. The levels of lipid metabolites in untreated control cells as referred to 1. Data are presented as a mean ± maximum semidispersion (*n* = 2)

	Control	TRIEN 48 h
Sphingolipids	1	2.50 ± 1.26
Pool of pospholipids	1	2.11 ± 0.05
Triacylglycerol	1	1.28 ± 0.03
Poly-unsaturated Fatty Acid (PUFA)	1	1.76 ± 0.10
Mono-unsaturated Fatty Acid (MUFA)	1	2.16 ± 0.39
Pool of Fatty Acyl chain (FA) determined at 1.6 ppm	1	1.88 ± 0.47
Total Cholesterol	1	1.57 ± 0.40

In addition, to test the effect on cell metabolism of TGFβ treatment, alone and following prolonged Cu depletion, we analyzed both the intracellular and the extracellular metabolome of MDA-MB-231. The intracellular levels of lactate were lower (about 17%) in TRIEN treated cells, alone and in combination with TGFβ, likely due to an enhanced efflux in the extracellular medium, as confirmed by the extracellular metabolome analysis results (Fig. 7a, right panel and Table 4), suggesting a shift into glycolysis in all protracted TRIEN treated cells. Furthermore, we found that TRIEN decreased the levels of metabolites involved in the cell energetic status such as (ATP + ADP) and (NAD + NADP), increased the level of formic acid (about 50%) and decreased (by 20%) choline phospholipids (tCho) contents, suggesting a metabolic reprogramming in these cells (Fig. 7a, right panel, and Table 4).

**Table 4:** NMR: Relative quantification (% metabolite/all metabolites) of aqueous metabolites involved in the main pathways detectable by NMR spectroscopy (9.4 T), in polar fraction of MDA-MB-231 cells (*n* = 2), following TGFβ treatment, alone and in cells pre-treated with TRIEN up to 6 days. Abbreviations: Cho = free choline; GPC = glycerophosphocoline; PCho = phosphocholine. Data are presented as mean ± maximum semidispersion (*n* = 2)

Metabolism	Metabolite	Control	TGFβ	TRIEN	TRIEN + TGFβ
Glucose/pyruvate metabolism	Lactic acid	39.52 ± 0.21	37.36 ± 4.73	33.13 ± 3.19	33.05 ± 1.12
One carbon metabolism	Formic acid	10.89 ± 3.41	10.61 ± 1.80	17.28 ± 5.22	15.20 ± 1.79
Nucleotide metabolism	ATP + ADP	1.23 ± 0.08	1.53 ± 0.24	0.61 ± 0.06	1.08 ± 0.22
	NAD + NADP	0.40 ± 0.09	0.90	0.32	0.29 ± 0.07
Aminoacids metabolism	L-phenylalanine	5.15 ± 0.67	5.82 ± 1.02	5.63 ± 0.25	6.31 ± 0.84
	L-tyrosine	1.26 ± 0.19	1.53 ± 0.35	1.16 ± 0.14	1.53 ± 0.12
	L-histidine	0.14 ± 0.11	0.24 ± 0.18	0.11 ± 0.02	0.21 ± 0.04
	L-glycine	2.62 ± 0.24	2.58 ± 0.09	2.21 ± 0.25	2.90 ± 0.06
	L-glutamic acid	6.26 ± 0.38	5.84 ± 0.30	6.83 ± 0.02	6.84 ± 0.52
	L-glutamine	1.94 ± 0.43	2.36 ± 0.16	2.45 ± 0.11	2.69 ± 0.11
	L-isoleucine	0.96 ± 0.01	1.32 ± 0.17	0.94 ± 0.03	1.12 ± 0.01
	L-aspartic acid	1.12 ± 0.49	1.64 ± 0.40	1.22 ± 0.11	1.52 ± 0.19
	L-alanine	3.38 ± 0.17	3.55 ± 0.02	3.41 ± 0.29	3.73 ± 0.12
Redox balance metabolism	Glutathione	3.61 ± 0.44	3.11 ± 0.10	3.55 ± 0.09	3.15 ± 0.10
	Taurine	2.32 ± 0.33	2.92 ± 0.34	2.38 ± 0.13	2.48 ± 0.22
Lipid metabolism	GPC + PCho + Cho	7.11 ± 0.21	6.17 ± 0.69	5.73 ± 0.38	4.74 ± 0.22
	Myo-inosytol	5.32 ± 0.20	4.91 ± 0.22	5.45 ± 0.13	4.97 ± 0.33
Lipid and aminoacid metabolism	Acetic acid	1.92 ± 0.26	2.05 ± 0.45	2.58 ± 0.38	2.42 ± 0.22
Krebs cycle	Succinic acid	0.37 ± 0.03	0.34 ± 0.03	0.32 ± 0.01	0.32 ± 0.02
Ornithine cycle	Polyamines	4.46 ± 0.72	5.66 ± 0.68	4.83 ± 0.27	5.45 ± 0.44

Then, the lipid intracellular metabolome was investigated in long-term treated cells as compared to untreated cells. We found that Cu depletion (alone and in combination with TGFβ) induced an accumulation of the pool of neutral and polar lipids, while this behavior was not observed in cells treated exclusively with TGFβ (Fig. 7b, right panel and Table 5), further suggesting a peculiar metabolic reprograming in TRIEN treated cells (Fig. 7b, right panel and Table 5).

**Table 5:** NMR: Relative quantification (Integral/1 × 10^6^ cells) of lipid metabolites involved in the main pathways detectable by NMR spectroscopy (14 T), in organic fraction, of MDA-MB-231 cells (*n* = 2), following 48-h treatment with TRIEN. The levels of lipid metabolites in untreated control cells as referred to 1. Data are presented as a mean ± maximum semidispersion (*n* = 2)

	Control	TGFβ	TRIEN	TRIEN + TGFβ
Sphingolipids	1	1.27 ± 0.66	1.38 ± 0.09	2.25 ± 0.61
Pool of phospholipids	1	0.91 ± 0.29	1.16 ± 0.36	1.36 ± 0.36
Triacylglycerol	1	0.84 ± 0.05	1.17 ± 0.14	1.31 ± 0.28
Poly-unsaturated Fatty Acids (PUFA)	1	0.93 ± 0.15	1.15 ± 0.36	1.22 ± 0.51
Mono-unsaturated Fatty Acids (MUFA)	1	0.86 ± 0.24	1.78 ± 0.86	1.54 ± 0.98
Pool of Fatty Acyl chain (FA) determined at 1.6 ppm	1	0.73 ± 0.05	1.45 ± 0.41	1.97 ± 0.67
Total Cholesterol	1	0.88 ± 0.35	1.23 ± 0.47	1.30 ± 0.63

The analysis of the extracellular metabolites, linked to pyruvate/glucose metabolism, revealed that prolonged exposure of the cells to TRIEN (6 days) (alone or in combination with TGFβ) determined a lower consumption and uptake of glucose than untreated and TGFβ treated cells (Fig. 7c). Indeed, TGFβ exposure did not counteract the minor consumption of glucose. On the other hand, both TRIEN and TGFβ treatments induced an accumulation of lactic acid and alanine in the extracellular medium of TNBC cells (Fig. 7c).

### Bioinformatics analyses positively correlate the survival of breast cancer patients with total and phosphorylated AKT and with CCS levels

**Fig. 8:**
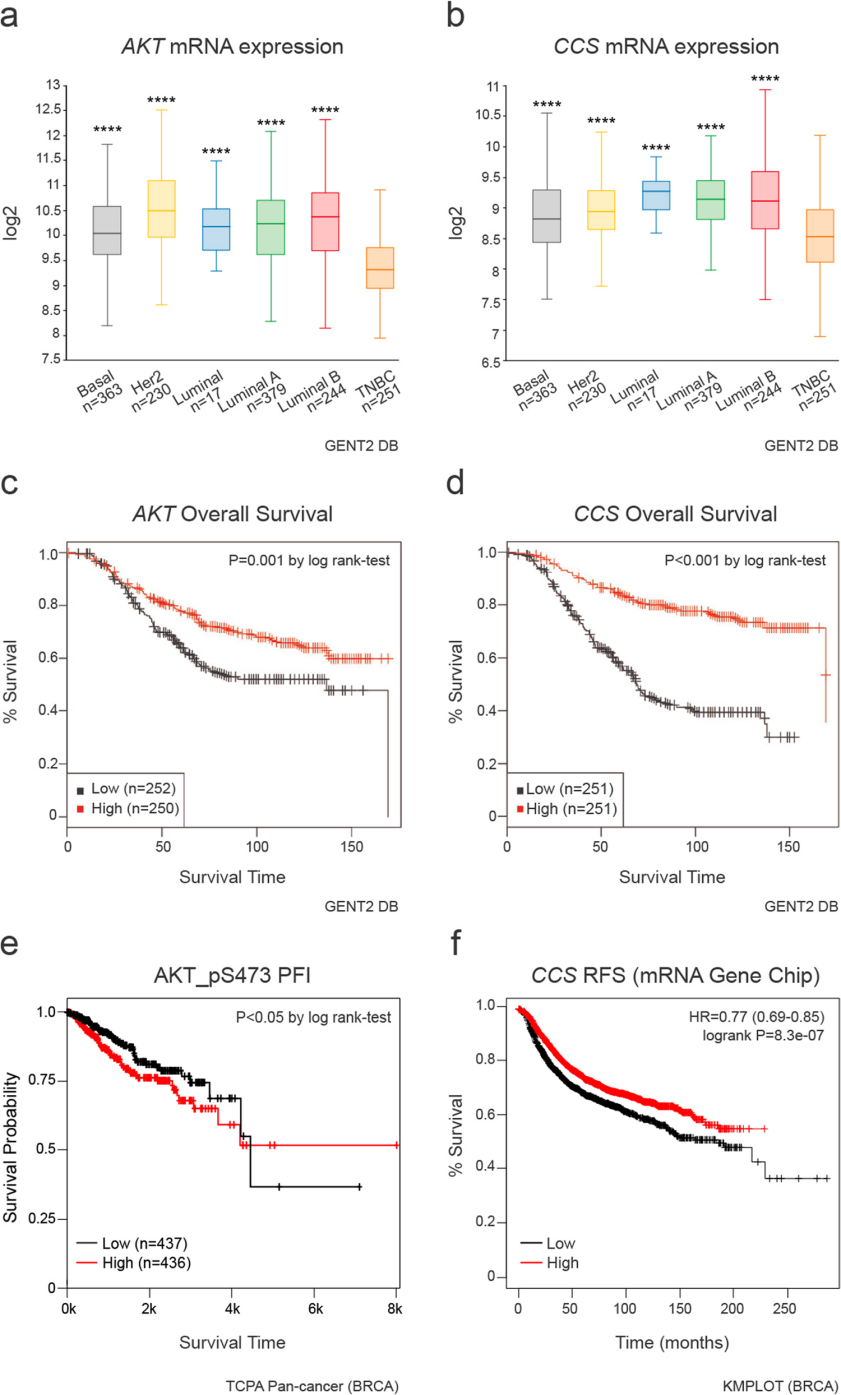
Database analysis correlates mRNA expression profile of AKT and CCS in TNBC patients to their survival. (**a**) AKT and (**b**) CCS mRNAs expression across various classes of breast cancer were retrieved from the Gene Expression Database of Normal and Tumor Tissues 2 (GENT2). Statistical significance was determined by one-way ANOVA. Kaplan–Meier overall survival (OS) plots comparing breast cancer patients with high and low mRNA levels of (**c**) AKT and (**d**) CCS obtained from GENT2. The survival between high- and low-mRNA levels cohorts was compared using log-rank tests. (**e**) Kaplan–Meier Progression Free Survival (PFI) plot comparing breast cancer patients with high and low level of phosphorylated AKT at Ser473 (AKT_pS473) recovered from The Cancer Proteome Atlas (TCPA) using the TCGA breast invasive carcinoma cohort (BRCA). (**f**) Relapse Free Survival (RFS) plot comparing breast cancer patients with high and low level of CCS mRNA levels retrieved from the Kaplan Meier Plotter (KMPLOT) database. The survival between high and low levels cohorts was compared using log-rank test

The Gene Expression Database of Normal and Tumor Tissues 2 (GENT2) was examined for *AKT* and *CCS* expression in patients affected by different subtypes of breast cancer, and in relation with their overall survival (OS) (Fig. 8a-d). Of note, TNBC patients showed lower expression of both *AKT* (Fig. 8a) and *CCS* (Fig. 8b) compared to the other investigated subtypes. Interestingly, we observed the same expression profile of *AKT* and *CCS* mRNAs also in the claudin low-breast cancer, characterized by the lack of HER2 and the expression of EMT genes (Figure S5). Moreover, the analysis of the OS showed that patients with higher levels of both *AKT* (Fig. 8c) and *CCS* (Fig. 8d) mRNAs were associated to a better outcome. Interestingly, the Progression Free Survival (PFI) plot for phosphorylated AKT [AKT_pS473, The Cancer Proteome Atlas (TCPA), using the TCGA breast invasive carcinoma cohort (BRCA)] and the Relapse Free Survival (RFS) plot for *CCS* mRNA (Kaplan Meier Plotter, KMPLOT database) [33], showed that the progression of the disease was significantly reduced in patients with higher levels of AKT activation (Fig. 8e) and with increased level of *CCS* (Fig. 8f), which, in turn implies a lower Cu content in those patients.

## Discussion

Deranged homeostasis of Cu has been indicated to be involved in several aspects of cell growth and differentiation, and therefore also in cancer. Besides the well-established role of this transition metal as cofactor of cuproenzymes, essential for metabolism, extracellular matrix organization and blood vessels development, more recently an intriguing role of Cu in modulating the activity of some kinases, especially those involved in cell growth, proliferation and autophagy, *i.e.*, mitogen-activated protein kinase 1/2 (MEK1/2) and the kinases ULK1 and ULK2, has been postulated. These findings have led to an extension of the concept of the essentiality of this metal for cell metabolism and to the importance of the preservation of its homeostasis. In fact, a recent and stimulating publication [34] formulated the concept of "cuproplasia" to attribute to this metal a fundamental role in cell growth processes related to cancer and also to extend its role far beyond the mere participation in the catalytic activity of established cuproenzymes in cell metabolism. This hypothesis may have a powerful relapse on cancer therapy, also because the control over Cu levels and reactivity is already an approved pharmacological approach for the treatment of the most common genetic disease associated with Cu overload, *i.e.*, Wilson’s disease, thus representing a good example of drug re-tasking.

In the present report, we have reduced the bioavailability of Cu in breast cancer cells by a specific Cu-chelator, TRIEN, one of the molecules currently used in Wilson's disease therapy [46] and in cancer clinical trials [24]. The aim of this study was to unravel a possible role of Cu in a specific phase of cancer cells spreading, the epithelial to mesenchymal transition (EMT). In fact, it has been shown that Cu depletion does not affect primary tumor mass, but it prevents the formation of metastases, as in lung cancer [47].

To this aim, we deliberately chose different breast cancer cells as tumor models, carrying different molecular features. Interestingly, it has been noted that EMT markers are more frequently expressed in TNBC compared with other breast cancer types [48, 49], though it has been shown a strong reduction of E-cadherin preferentially in the Luminal A/Luminal B subtypes [50].

Our results demonstrate that TNBC cells (*i.e.,* MDA-MB-231 and SUM159) and HER2 overexpressing cells (*i.e.,* SK-BR-3) have a more basal mesenchymal phenotype than the luminal A cells, T47D, which rather showed a mixed phenotype, expressing at high level both the epithelial marker E-cadherin and the mesenchymal one αSMA. We also compare the level of the main Cu-transporters/chaperones and cuproenzymes in all cell lines. We found that MDA-MB-231 is the TNBC cell line with the highest expression of the Cu pump ATP7A, of the Cu Chaperone for Superoxide Dismutase (CCS) and the only one to express, at appreciable levels, the cuproenzyme LOXL2 involved in the extracellular matrix remodeling, thus suggesting a greater Cu availability in this cell line in comparison to the others. Of note, these differences seem not to be relevant in our experimental design. Treatment with the specific Cu-chelator TRIEN demonstrated to be efficient in decreasing Cu bioavailability in all cell subtypes, since Cu-dependent markers showed the expected response (*i.e.,* CCS level increased, SOD1 activity decreased as well as the level of the subunit II of cytochrome c oxidase). However, TRIEN treatment lead to the identification of two distinct cancer cell subtypes: Triple Negative (*i.e.,* MDA-MB-231 and SUM159) and the luminal A (*i.e.,* T47D) cells *versus* HER2 overexpressing cells (*i.e.,* SK-BR-3). Notably, in the first group, Cu depletion is achieved within 24 h treatment, and it drives cells towards a more mesenchymal and thus aggressive phenotype (increase in fibronectin/αSMA levels and decrease in E-cadherin levels accompanied by an increased extent of invasiveness, as shown in the migration and wound healing assays). On the contrary, in SK-BR-3 Cu depletion is obtained later, after 48 h, and it forces cells towards a more epithelial and less aggressive phenotype. Furthermore, the differential behavior of HER2 negative cells and of HER2-overexpressing cells is undoubtedly dependent on Cu bioavailability, because the addition of exogenous Cu, as CuSO_4_, reversed the effects on EMT markers in all cell types tested. Of note, these results are supported by preclinical models in which it has been shown that another Cu-chelator, TTM, suppresses lung metastases without affecting primary breast tumors [47]. Indeed, in our experiments, treatment with TTM showed superimposable results on TRIEN, at least in terms of migratory capacity of cells.

To deepen our investigation, we analyzed the possible signaling involved in the induction of EMT following Cu depletion. Interestingly, upon TRIEN treatment (both 24 h and 6 days) we observe a persistent activation of the AKT/GSK3β/SNAIL axis in all the cell lines with the exception, again, of SK-BR-3. These data fit perfectly with the expression of mesenchymal markers, and with the transcriptional repression of the E-cadherin gene in MDA-MB-231, SUM159 and T47D related to the stabilization of the transcription factor SNAI1 [51]. By contrast, in SK-BR-3 cells Cu depletion results in a less aggressive and less mesenchymal phenotype, in accordance with the lack of AKT activation. In particular, we detected the phosphorylation of AKT at Ser473 residue, but not at Thr308 residue. The lack of phosphorylation of the Thr308 residue can be easily explained considering the mechanism required for AKT activation, involving the Cu-dependent kinase PDK1 [52]. Indeed, it has been shown that the activation of PDK1, in Cu adequate condition, relies on the high affinity Cu transporter CTR1. Thus, the induction of Cu deficiency could impair PDK1 activity.

It has been demonstrated that, in breast cancer, LOXL2 promotes angiogenesis through the activation of the AKT-SNAI1 and ERK pathways [53]. However, upon Cu depletion, we observed the activation of AKT, despite the reduced levels of LOXL2. Therefore, in TNBC cells our results suggest the requirement of a Cu-dependent modulation of cell aggressiveness by a mechanism completely different from that already described which involves CTR1 and LOXL2. In addition, the PDK1/AKT signaling cascade proceed following the homo/heterodimerization of HER2 receptor [54] lacking in our TNBC (MDA-MB-231 and SUM159) and luminal A (T47D) cellular models, further confirming the “alternative” route of AKT regulation driven by Cu bioavailability. Thus, we could postulate that the maintenance of Cu homeostasis is necessary for PDK1 activity, which in turn promotes the phosphorylation of AKT at Thr308 but prevents that occurring at Ser473. Additionally, we can also hypothesize that Cu may affect another tyrosine kinase receptor (TKR) or a G-protein-coupled receptor, modulating AKT signaling cascade. To validate this hypothesis, further studies are underway in our laboratory to better characterize the involvement of Cu in this unconventional EMT signal cascade, which also triggers the acquisition of drug resistance [55].

Considering these results, we evaluated whether perturbation of Cu bioavailability could affect the induction of EMT promoted by TGFβ. Indeed, it is well known that the increased secretion of TGFβ in the tumor microenvironment is one of the main events underlying EMT and necessary for cancer cells spreading [54, 56, 57]. Thus, we depleted our breast cancer cell models of Cu, for at least 5 days, and then we treated them with TGFβ, for additional 24 h, always keeping cells in the presence of TRIEN. As expected, TGFβ treatment induced the acquisition of mesenchymal tracts in all cell lines analyzed, including in SK-BR-3. These results were superimposable on those obtained in MDA-MB-231, SUM159 and T47D after 24 h of Cu depletion, further supporting the acquisition of a mesenchymal phenotype by these cells following perturbation of Cu homeostasis. Furthermore, the exposure of MDA-MB-231 and SUM159 cells to TGFβ alone resulted in the increase of proteins involved in ECM remodeling (*i.e.*, MMP2/MMP9, LOXL2 and fibronectin). Conversely, in Cu deficiency, TGFβ was no longer able to induce EMT. Indeed, we observed a drastic reduction of the epithelial marker E-cadherin in MDA-MB-231 and even an increase in its level in the SK-BR-3 and SUM159 cell lines, parallel to a drastic drop in mesenchymal markers (fibronectin and αSMA) in all cell lines. Furthermore, we no longer observed the activation and secretion of enzymes involved in remodeling the ECM. The reduction in the bioavailability of Cu is the determining factor in allowing TGFβ to induce EMT, as the same results were obtained in cells treated for 6 days with TRIEN alone. Indeed, beside the persistent activation of AKT, we found also that Cu depletion impairs the TGFβ induced activation of STAT3. Of note, it has been recently shown the involvement of a Cu-dependent amine oxidase in the activation of the IL6/JAK/STAT3 axis required for the progression of hepatocellular carcinoma [58].

The relevance of our findings on the relation between Cu homeostasis and the activation of AKT in modulating TNBC aggressiveness was further corroborated by the analysis of the GENT2 DB, TCPA Pan-cancer (BRCA) and KMPLOT databases of the mRNA level of total *AKT* and *CCS*, pAKT-S473 in breast cancer patients. Intriguingly, we found that TNBC patients show lower levels of both total *AKT* and *CCS* than patients affected by other tumor subtypes.We found the same expression profile also in the claudin-low breast tumors, aggressive subtypes of highly heterogeneous malignancies characterized by the lack of HER2, the low expression of cell–cell adhesion genes and high expression of EMT genes [59]. Of note, greater survival outcomes occur in patients with higher levels of both total and phosphorylated AKT and with higher levels of *CCS*, implying lower Cu content, further strengthening the significance of Cu homeostasis-mediated modulation of the AKT signaling observed in our experimental models and affecting TNBC spreading.

The results obtained by the metabolomic approach shown in the present paper confirms what previously reported. Indeed, in a recent paper by Ramchadani et al*.* (2021) Cu depletion by TTM has been demonstrated to modulate mitochondrial oxidative phosphorylation, increased extracellular levels of lactic acid and impair TNBC metastasis [60]. Furthermore, in the study by Ishida et al. (2013) has been reported that the reduction of systemic Cu with a chelating drug impaired mitochondrial energy metabolism and decreased ATP levels, despite induction of glycolysis, a phenomenon not accompanied by an increased invasiveness of tumors in vivo experimental models [41]. In our experimental model, exposure to TRIEN lead to a strong reduction of the mitochondrial energy metabolism of TNBC cells, and to a shift towards glycolysis, accompanied by the extracellular increase of lactate. The alteration of oxidative metabolism would also explain the reduction we observe in the formation of acetyl CoA, accompanied by the accumulation of its precursors (acetate and CO_2_) and an interesting increase in the levels of formate (precursor of nucleotides).

During EMT cell morphology change is accompanied by a profound rearrangement of cell membrane fluidity due to modification in lipid composition; thus there is a great attention to lipid metabolism in cancer [61]. Indeed, abnormal lipid metabolism goes together with invasion and metastasis; specifically, proliferation of cancer cells requires both increased lipid synthesis and decreased breakdown. However, the effect of TGFβ on cancer cells lipids metabolism during EMT is controversial [61]. Our results confirm that TRIEN treatment of TNBC cells, both for short- and long-term, alone or in combination with TGFβ, led to an accumulation of both polar and neutral lipids, in opposition with the data obtained with TGFβ alone. Thus, again TRIEN counteracts TGFβ effect also on the lipidome.

## Conclusions

**Fig. 9:**
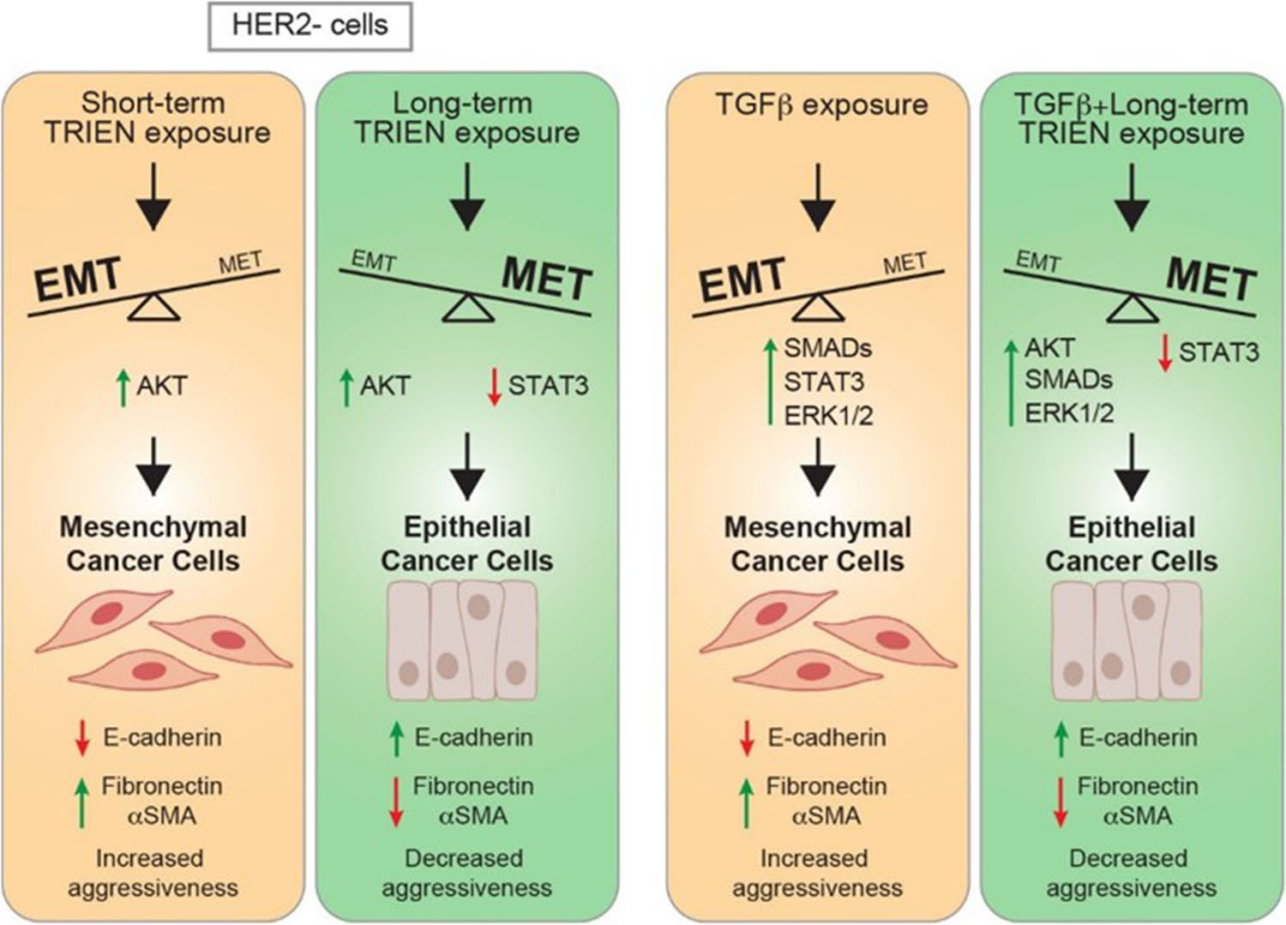
Graphical representation of the hypothetical mechanisms proposed for the regulation by copper of proteins/kinases involved in Her2 negative and Her2-overexpressing breast cancer cells aggressiveness

In conclusion, the results obtained in the present study pinpoint a different response of the EMT hallmarks to the short- and long-term exposure to TRIEN, pushing, at first, HER2 negative cells towards a more aggressive behavior and, upon prolonged treatment, strengthening their epithelial features, thus reducing their invasiveness. A graphical representation of the hypothetical mechanisms undergoing and of the proteins/kinases involved is shown in Fig. 9. This phenomenon may be related to the different impact of the short and prolonged activation of the AKT kinase and to the repression of STAT3 signaling, but also to an initial mechanism of resistance to the treatment itself, which disappears with the chronic exposure to the chelator. Indeed, many studies have demonstrated the correlation between the activation of the EMT program and the acquisition of cell resistance to chemio-, radio- and immuno-therapy. Furthermore, even the therapy itself seems to favor the activation of EMT and, therefore, the selection of cells with a more aggressive phenotype [62, 63].

However, further investigation is required to dissect the possible modulation of AKT driven by intracellular Cu levels. Indeed, we will investigate the possible role played by Cu in regulating the secretion of growth factors that trigger the EMT program (e.g. TGFβ) or in the activation of RTK or G-coupled receptors. These studies will shed light on identifying novel agents involved in the main pathways underlying the spread of TNBC which can be targeted by therapy.

## Supplementary Information

Below is the link to the electronic supplementary material.

Additional Figure 1 (PDF 388 KB) **Effect of TRIEN treatment on breast cancer cell survival.** Cells were exposed to different concentration of TRIEN (ranging from 2 to 1000 μM) for 72 h and cell viability assessed by MTS assay.** (a) **MDA-MB-231; **(b) **SUM 159; **(c) **T47D; **(d)** SK-BR-3.

Supplementary Figure 2 (PDF 730 KB)** Restoring copper bioavailability reverts the altered expression of epithelial to mesenchymal markers. **Cells were treated with 125 μM TRIEN for 24 (MDA-MB-231) or 48 h (SK-BR-3). Afterwards TRIEN was removed and cells incubated for 3 h with medium supplemented with 100 μM CuSO_4_. Western blot (**left panels**) and densitometric analyses (**right panels**) for fibronectin, E-cadherin, αSMA, CCS and the subunit II of cytochrome c oxidase are shown for **(a) **MDA-MB-231 and **(b) **SK-BR-3 cells. Twenty micrograms of proteins were loaded on each lane. Vinculin was used as loading control. One representative blot is shown for each antigen, data are presented as a mean ± SEM (n ≥ 3, ONE-way ANOVA *p < 0.05; ***p < 0.001 ****p < 0.0001).

Supplementary Figure 3 (PDF 5553 KB) **Prolonged TRIEN exposure counteracts TGFβ treatment impairing the extracellular release of fibronectin and altering cells morphology. **Western blot analysis of the level of extracellular release of fibronectin in **(a) **MDA-MB-231 and **(b)** SUM159, following 10 ng/ml TGFβ or 125 μM TRIEN treatment, for 24 h, alone and in combination. Ponceau S staining was used as loading control. **(c**, **left panels)** analysis of cell morphology and **(c**, **right panels) **cell circularity upon treatment with 10 ng/ml TGFβ alone or in combination with 125 μM TRIEN, for 24 h, were measured using the ImageJ software. One representative blot/image is shown; data are presented as a mean ± SEM (n ≥ 3, ONE-Way ANOVA, *p < 0.05, ***p < 0.001, ****p < 0.0001). Calibration bar corresponds to 100 μm.

Supplementary Figure 4 (JPG 973 KB) **TRIEN pretreatment modulates TGFβ effects in MDA-MB-231 cells. a) Cells were** treated with TGFβ (24 h) or 125 µM TRIEN (6 days), alone and in combination, Western blot (**left panel**) and densitometric analyses (**right panel**) for E-cadherin is shown. Twenty micrograms of proteins were loaded on each lane. GAPDH was used as loading control. One representative blot is shown for each antigen, data are presented as a mean ± SEM (n ≥ 3, ONE-way ANOVA, ***p < 0.001, ****p < 0.0001). **b)** Immunofluorescence images of fibronectin (left panels) and their corresponding signal analysis (right panel). Calibration bar: 100 μm. 40 × magnification. One representative image is shown; data are presented as a mean ± SEM (n ≥ 3, ONEway ANOVA, **p < 0.01 *vs* vehicle, ***p < 0.001 *vs* vehicle,^###^p < 0.001, vs TGFβ).

Supplementary Figure 5 (JPG 891 KB) **Claudin-low breast cancers are characterized by low levels of mRNA of *HER2*, *AKT* and *CCS* in comparison to other breast cancer subtypes. (a)** HER2, **(b) **AKT and **(c)** CCS mRNAs expression levels across various classes of breast cancer were retrieved from The Cancer Genome Atlas Program (TCGA) database by the cBioPortal For Cancer Genomic tool. Dots in different colors represent the breast cancer histological grade of each patient included in the analysis: red for the first neoplasm histological grade, yellow for the second neoplasm histological grade and blue for the third neoplasm histological grade. In **(a)** the percentage of patients affected by the different histological grade is also reported for each subclass of breast cancer.

## Data Availability

The raw data obtained and analyzed during the current study is available from the corresponding authors on reasonable request**.**

## Abbreviations

ATP7A: ATPase Copper Transporting Alpha
EMT: Epithelial to mesenchymal transition
Cu: Copper
TRIEN: Triethylenetetramine
TTM: Tetrathiomolybdate
SOD1: Cu-dependent Superoxide dismutase
CCS: Copper chaperone for superoxide dismutase 1
αSMA: Alpha Smooth Muscle Actin
AKT: Protein kinase B, PKB
GSK3β: Glycogen synthase kinase-3 beta
MEK1/2: MAP kinase kinase
ERK1/2: Extracellular signal-regulated kinases
ULK1/2: Unc-51 like autophagy activating kinase
TGFβ: Transforming growth factor beta
LOXL2: Lysyl Oxidase Like 2
HER2: Human Epidermal Growth Factor Receptor 2
MMP2/9: Matrix Metalloproteinase 2/9
TNBC: Triple Negative Breast Cancer
STAT3: Signal Transducer and Activator of Transcription 3
ER: Estrogen Receptor
PR: Progesteron Receptor
ECM: Extracellular Matrix
SMAD1/2: Small Mother Against Decapentaplegic

## References

[1] Zubair M, Wang S, Ali N (2020). Advanced Approaches to Breast Cancer Classification and Diagnosis. Front. Pharmacol..

[2] A. De Luca, A. Barile, M. Arciello, L. Rossi, Copper Homeostasis as Target of Both Consolidated and Innovative Strategies of Anti-Tumor Therapy. J. Trace Elem. Med. Biol. 55 (2019). 10.1016/j.jtemb.2019.06.008 10.1016/j.jtemb.2019.06.008 31345360

[3] C.Y. Loh, J.Y. Chai, T.F. Tang, W.F. Wong, G. Sethi, M.K. Shanmugam, P.P. Chong, C.Y. Looi, The E-Cadherin and N-Cadherin Switch in Epithelial-to-Mesenchymal Transition: Signaling, Therapeutic Implications, and Challenges. Cells. 8 (2019). 10.3390/cells8101118 10.3390/cells8101118 PMC6830116 31547193

[4] Dongre A, Weinberg RA (2019). New Insights into the Mechanisms of Epithelial-Mesenchymal Transition and Implications for Cancer. Nat. Rev. Mol. Cell Biol..

[5] Deshmukh AP, Vasaikar SV, Tomczak K, Tripathi S, Den Hollander P, Arslan E, Chakraborty P, Soundararajan R, Jolly MK, Rai K (2021). Identification of EMT Signaling Cross-Talk and Gene Regulatory Networks by Single-Cell RNA Sequencing. Proc. Natl. Acad. Sci. U. S. A..

[6] G.V. Vijay, N. Zhao, P. Den Hollander, M.J. Toneff, R. Joseph, M. Pietila, J.H. Taube, T.R. Sarkar, E. Ramirez-Pena, S.J. Werden et al., GSK3β Regulates Epithelial-Mesenchymal Transition and Cancer Stem Cell Properties in Triple-Negative Breast Cancer. Breast Cancer Res. 21 (2019). 10.1186/s13058-019-1125-0 10.1186/s13058-019-1125-0 PMC6407242 30845991

[7] Brabletz S, Schuhwerk H, Brabletz T, Stemmler MP (2021). Dynamic EMT: A Multi-Tool for Tumor Progression. EMBO J..

[8] O. Repetto, P. De Paoli, V. De Re, V. Canzonieri, R. Cannizzaro, Levels of Soluble E-Cadherin in Breast, Gastric, and Colorectal Cancers. Biomed Res. Int. 2014 (2014). 10.1155/2014/408047. 10.1155/2014/408047 PMC4182303 25535613

[9] McGuire JK, Li Q, Parks WC (2003). Matrilysin (Matrix Metalloproteinase-7) Mediates E-Cadherin Ectodomain Shedding in Injured Lung Epithelium. Am. J. Pathol..

[10] D.C. Radisky, D.D. Levy, L.E. Littlepage, H. Liu, C.M. Nelson, J.E. Fata, D. Leake, E.L. Godden, D.G. Albertson, M.A. Nieto et al., Rac1b and Reactive Oxygen Species Mediate MMP-3-Induced EMT and Genomic Instability. (2005). 10.1038/nature03688 10.1038/nature03688 PMC2784913 16001073

[11] Denoyer D, Masaldan S, La Fontaine S, Cater MA (2015). Targeting Copper in Cancer Therapy: “Copper That Cancer”. Metallomics.

[12] Shanbhag VC, Gudekar N, Jasmer K, Papageorgiou C, Singh K, Petris MJ (2021). Copper Metabolism as a Unique Vulnerability in Cancer. Biochim. Biophys. acta. Mol. cell Res..

[13] S.C. Dinca, D. Greiner, K. Weidenfeld, L. Bond, D. Barkan, C.L. Jorcyk, Novel Mechanism for OSM-Promoted Extracellular Matrix Remodeling in Breast Cancer: LOXL2 Upregulation and Subsequent ECM Alignment. Breast Cancer Res. 23 (2021). 10.1186/s13058-021-01430-x 10.1186/s13058-021-01430-x PMC8132418 34011405

[14] Sorokin AV, Chen J (2013). MEMO1, a New IRS1-Interacting Protein, Induces Epithelial&ndash;Mesenchymal Transition in Mammary Epithelial Cells. Oncogene.

[15] Grossman M, Ben-Chetrit N, Zhuravlev A, Afik R, Bassat E, Solomonov I, Yarden Y, Sagi I (2016). Tumor Cell Invasion Can Be Blocked by Modulators of Collagen Fibril Alignment That Control Assembly of the Extracellular Matrix. Cancer Res..

[16] Schotanus MD, Van Otterloo E (2020). Finding Memo—Emerging Evidence for Memo1′ s Function in Development and Disease. Genes (Basel)..

[17] Blockhuys S, Celauro E, Hildesjö C, Feizi A, Stål O, Fierro-González JC, Wittung-Stafshede P (2017). Metallomics Defining the Human Copper Proteome and Analysis of Its Expression Variation in Cancers †. Metallomics.

[18] Chen G-F, Sudhahar V, Youn S-W, Das A, Cho J, Kamiya T, Urao N, McKinney RD, Surenkhuu B, Hamakubo T (2015). Copper Transport Protein Antioxidant-1 Promotes Inflammatory Neovascularization via Chaperone and Transcription Factor Function. Sci. Rep..

[19] S. Blockhuys, X. Zhang, P. Wittung-Stafshede, Single-Cell Tracking Demonstrates Copper Chaperone Atox1 to Be Required for Breast Cancer Cell Migration. 10.1073/pnas.1910722117/-/DCSupplemental 10.1073/pnas.1910722117 PMC6995000 31932435

[20] Jana A, Das A, Krett NL, Guzman G, Thomas A, Mancinelli G, Bauer J, Ushio-Fukai M, Fukai T, Jung B (2020). Nuclear Translocation of Atox1 Potentiates Activin A-Induced Cell Migration and Colony Formation in Colon Cancer. PLoS ONE.

[21] M.L. Turski, D.C. Brady, H.J. Kim, B.-E. Kim, Y. Nose, C.M. Counter, D.R. Winge, D.J. Thiele, A Novel Role for Copper in Ras/Mitogen-Activated Protein Kinase Signaling. (2012). 10.1128/MCB.05722-11 10.1128/MCB.05722-11 PMC3302449 22290441

[22] Tsang T, Posimo JM, Gudiel AA, Cicchini M, Feldser DM, Brady DC (2020). Copper Is an Essential Regulator of the Autophagic Kinases ULK1/2 to Drive Lung Adenocarcinoma. Nat. Cell Biol..

[23] Jain S, Cohen J, Ward MM, Kornhauser N, Chuang E, Cigler T, Moore A, Donovan D, Lam C, Cobham MV (2013). Tetrathiomolybdate-Associated Copper Depletion Decreases Circulating Endothelial Progenitor Cells in Women with Breast Cancer at High Risk of Relapse. Ann. Oncol. Off. J. Eur. Soc. Med. Oncol..

[24] S. Baldari, G. Di Rocco, G. Toietta, Current Biomedical Use of Copper Chelation Therapy. Int. J. Mol. Sci. **21** (2020). 10.3390/ijms21031069 10.3390/ijms21031069 PMC7037088 32041110

[25] L. Rossi, M.F. Lombardo, M.R. Ciriolo, G. Rotilio, Mitochondrial Dysfunction in Neurodegenerative Diseases Associated with Copper Imbalance. Neurochem Res. **29, **493–504(2004). 10.1023/B:NERE.0000014820.99232.8a 10.1023/b:nere.0000014820.99232.8a 15038597

[26] Gismondi A, Nanni V, Reina G, Orlanducci S, Terranova ML, Canini A (2016). Nanodiamonds Coupled with 5,7-Dimethoxycoumarin, a Plant Bioactive Metabolite, Interfere with the Mitotic Process in B16F10 Cells Altering the Actin Organization. Int. J. Nanomedicine.

[27] E. Saulle, I. Spinello, M.T. Quaranta, L. Pasquini, E. Pelosi, E. Iorio, G. Castelli, M. Chirico, M.E. Pisanu, T. Ottone, et al., Targeting Lactate Metabolism by Inhibiting MCT1 or MCT4 Impairs Leukemic Cell Proliferation, Induces Two Different Related Death-Pathways and Increases Chemotherapeutic Sensitivity of Acute Myeloid Leukemia Cells. Front. Oncol. 10 (2021). 10.3389/FONC.2020.621458/FULL 10.3389/fonc.2020.621458 PMC7892602 33614502

[28] S.-J. Park, B.-H. Yoon, S.-K. Kim, S.-Y. Kim, GENT2: An Updated Gene Expression Database for Normal and Tumor Tissues. 10.1186/s12920-019-0514-7 10.1186/s12920-019-0514-7 PMC6624177 31296229

[29] E. Cerami, J. Gao, U. Dogrusoz, B.E. Gross, S.O. Sumer, A. Aksoy, A. Jacobsen, C.J. Byrne, M.L. Heuer, E. Larsson et al., The CBio Cancer Genomics Portal: An Open Platform for Exploring Multidimensional Cancer Genomics Data. CANCER Discov. 401 (2012). 10.1158/2159-8290.CD-12-0095 10.1158/2159-8290.CD-12-0095 PMC3956037 22588877

[30] J. Gao, B.A. Aksoy, U. Dogrusoz, G. Dresdner, B. Gross, S.O. Sumer, Y. Sun, A. Jacobsen, R. Sinha, E. Larsson et al., Integrative Analysis of Complex Cancer Genomics and Clinical Profiles Using the CBioPortal. Sci. Signal. 6, pl1 (2013). 10.1126/scisignal.2004088 10.1126/scisignal.2004088 PMC4160307 23550210

[31] J. Li, R. Akbani, W. Zhao, Y. Lu, J.N. Weinstein, G.B. Mills, H. Liang, Focus on Computer Resources Explore, Visualize, and Analyze Functional Cancer Proteomic Data Using the Cancer Proteome Atlas. 10.1158/0008-5472.CAN-17-0369. 10.1158/0008-5472.CAN-17-0369 PMC5679242 29092939

[32] J. Li, Y. Lu, R. Akbani, Z. Ju, P.L. Roebuck, W. Liu, J.-Y. Yang, B.M. Broom, R.G.W. Verhaak, D.W. Kane et al., TCPA: A Resource for Cancer Functional Proteomics Data. (2013). 10.1038/nmeth.2650 10.1038/nmeth.2650 PMC4076789 24037243

[33] Győrffy B (2021). Survival Analysis across the Entire Transcriptome Identifies Biomarkers with the Highest Prognostic Power in Breast Cancer. Comput. Struct. Biotechnol. J..

[34] Ge EJ, Bush AI, Casini A, Cobine PA, Cross JR, DeNicola GM, Dou QP, Franz KJ, Gohil VM, Gupta S (2021). Connecting Copper and Cancer: From Transition Metal Signalling to Metalloplasia. Nat. Rev. Cancer.

[35] Rossi L, Marchese E, Lombardo MF, Rotilio G, Ciriolo MR (2001). Increased Susceptibility of Copper-Deficient Neuroblastoma Cells to Oxidative Stress-Mediated Apoptosis. Free Radic. Biol. Med..

[36] Bertinato J, L’Abbé MR (2003). Copper Modulates the Degradation of Copper Chaperone for Cu, Zn Superoxide Dismutase by the 26 S Proteosome. J. Biol. Chem..

[37] Capo CR, Pedersen JZ, Falconi M, Rossi L (2017). Oleuropein Shows Copper Complexing Properties and Noxious Effect on Cultured SH-SY5Y Neuroblastoma Cells Depending on Cell Copper Content. *J. **trace** Elem*. Med. Biol. Organ Soc. Miner. Trace Elem..

[38] da Silva DA, De Luca A, Squitti R, Rongioletti M, Rossi L, Machado CML, Cerchiaro G (2022). Copper in Tumors and the Use of Copper-Based Compounds in Cancer Treatment. J. Inorg. Biochem..

[39] G.J. Brewer, F. Askari, M.T. Lorincz, M. Carlson, M. Schilsky, K.J. Kluin, P. Hedera, P. Moretti, J.K. Fink, R. Tankanow et al., Treatment of Wilson disease with ammonium tetrathiomolybdate: IV. Comparison of tetrathiomolybdate and trientine in a double-blind study of treatment of the neurologic presentation of Wilson disease. Arch Neurol. **63**, 21–527 (2006). 10.1001/archneur.63.4.521 10.1001/archneur.63.4.521 16606763

[40] Ryumon S, Okui T, Kunisada Y, Kishimoto K, Shimo T, Hasegawa K, Ibaragi S, Akiyama K, Thu Ha NT, Monsur Hassan NM (2019). Ammonium Tetrathiomolybdate Enhances the Antitumor Effect of Cisplatin via the Suppression of ATPase Copper Transporting Beta in Head and Neck Squamous Cell Carcinoma. Oncol. Rep..

[41] Ishida S, Andreux P, Poitry-Yamate C, Auwerx J, Hanahan D (2013). Bioavailable Copper Modulates Oxidative Phosphorylation and Growth of Tumors. Proc. Natl. Acad. Sci..

[42] Hermida MA, Dinesh Kumar J, Leslie NR (2017). GSK3 and Its Interactions with the PI3K/AKT/MTOR Signalling Network. Adv. Biol. Regul..

[43] Beurel E, Grieco SF, Jope RS (2015). Glycogen Synthase Kinase-3 (GSK3): Regulation, Actions, and Diseases. Pharmacol. Ther..

[44] Xu J, Lamouille S, Derynck R (2009). TGF-β-Induced Epithelial to Mesenchymal Transition EMT: Loss of Epithelial and Acquisition of Mesen-Chymal Characteristics. Cell Res..

[45] Radisky ES, Radisky DC, Radisky ES, Radisky DC (2010). Matrix Metalloproteinase-Induced Epithelial-Mesenchymal Transition in Breast Cancer. J Mammary Gland Biol Neoplasia.

[46] Walshe JM (1982). Treatment of Wilson’s Disease with Trientine (Triethylene Tetramine) Dihydrochloride. Lancet (London, England).

[47] N. Chan, A. Willis, N. Kornhauser, M.M. Ward, S.B. Lee, E. Nackos, B.R. Seo, E. Chuang, T. Cigler, A. Moore et al., Cancer Therapy: Clinical Influencing the Tumor Microenvironment: A Phase II Study of Copper Depletion Using Tetrathiomolybdate in Patients with Breast Cancer at High Risk for Recurrence and in Preclinical Models of Lung Metastases. Clin Cancer Res. 23 (2017). 10.1158/1078-0432.CCR-16-1326 10.1158/1078-0432.CCR-16-1326 27769988

[48] Levin C, Jørgensen T, Forsare C, Bendahl P-O, Falck A-K, Fernö M, Lövgren K, Aaltonen K, Rydén L (2020). Expression of Epithelial-Mesenchymal Transition-Related Markers and Phenotypes during Breast Cancer Progression. Breast Cancer Res. Treat..

[49] Jeong H, Ryu Y, An J, Lee Y, Kim A (2012). Epithelial-Mesenchymal Transition in Breast Cancer Correlates with High Histological Grade and Triple-Negative Phenotype. Histopathology.

[50] V. Pomp, C. Leo, A. Mauracher, D. Korol, W. Guo, Z. Varga, Differential Expression of Epithelial-Mesenchymal Transition and Stem Cell Markers in Intrinsic Subtypes of Breast Cancer. Breast Cancer Res. Treat. 154. 10.1007/s10549-015-3598-6 10.1007/s10549-015-3598-6 26467042

[51] Liu Z-C, Wang H-S, Zhang G, Liu H, Chen X-H, Zhang F, Chen D-Y, Cai S-H, Du J (2014). AKT/GSK-3β Regulates Stability and Transcription of Snail Which Is Crucial for BFGF-Induced Epithelial-Mesenchymal Transition of Prostate Cancer Cells. Biochim. Biophys. Acta.

[52] J. Guo, J. Cheng, N. Zheng, X. Zhang, X. Dai, L. Zhang, C. Hu, X. Wu, Q. Jiang, D. Wu et al., Copper Promotes Tumorigenesis by Activating the PDK1-AKT Oncogenic Pathway in a Copper Transporter 1 Dependent Manner. (2021). 10.1002/advs.202004303 10.1002/advs.202004303 PMC8456201 34278744

[53] Wang C, Xu S, Tian Y, Ju A, Hou Q, Liu J, Fu Y, Luo Y (2019). Lysyl Oxidase-Like Protein 2 Promotes Tumor Lymphangiogenesis and Lymph Node Metastasis in Breast Cancer. Neoplasia.

[54] P. Gupta, S.K. Srivastava, HER2 Mediated de Novo Production of TGFb Leads to SNAIL Driven Epithelial-to-Mesenchymal Transition and Metastasis of Breast Cancer 5 (2014). 10.1016/j.molonc.2014.06.006 10.1016/j.molonc.2014.06.006 PMC4252481 24994678

[55] C. Dong, J. Wu, Y. Chen, J. Nie, C. Chen, Activation of PI3K/AKT/MTOR Pathway Causes Drug Resistance in Breast Cancer. 10.3389/fphar.2021.628690 10.3389/fphar.2021.628690 PMC8005514 33790792

[56] Pang M-F, Georgoudaki A-M, Lambut L, Johansson J, Tabor V, Hagikura K, Jin Y, Jansson M, Alexander JS, Nelson CM (2016). TGF-&beta;1-Induced EMT Promotes Targeted Migration of Breast Cancer Cells through the Lymphatic System by the Activation of CCR7&sol;CCL21-Mediated Chemotaxis. Oncogene.

[57] Y. Hao, D. Baker, P. Ten Dijke, Molecular Sciences TGF-β-Mediated Epithelial-Mesenchymal Transition and Cancer Metastasis. 10.3390/ijms20112767. 10.3390/ijms20112767 PMC6600375 31195692

[58] Q. Ding, D. Lin, Y. Zhou, F. Li, J. Lai, J. Duan, J. Chen, C. Jiang, Downregulation of Amine Oxidase Copper Containing 1 Inhibits Tumor Progression by Suppressing IL-6/JAK/STAT3 Pathway Activation in Hepatocellular Carcinoma. Oncol. Lett. 22 (2021). 10.3892/ol.2021.13118 10.3892/ol.2021.13118 PMC8581477 34777591

[59] R.M. Pommier, A. Sanlaville, L. Tonon, J. Kielbassa, E. Thomas, A. Ferrari, A.-S. Sertier, F. Hollande, P. Martinez, A. Tissier et al., Comprehensive Characterization of Claudin-Low Breast Tumors Reflects the Impact of the Cell-of-Origin on Cancer Evolution. 10.1038/s41467-020-17249-7 10.1038/s41467-020-17249-7 PMC7347884 32647202

[60] D. Ramchandani, M. Berisa, D.A. Tavarez, Z. Li, M. Miele, Y. Bai, S.B. Lee, Y. Ban, N. Dephoure, R.C. Hendrickson et al., Copper Depletion Modulates Mitochondrial Oxidative Phosphorylation to Impair Triple Negative Breast Cancer Metastasis. Nat. Commun. 12 (2021). 10.1038/S41467-021-27559-Z 10.1038/s41467-021-27559-z PMC8674260 34911956

[61] W. Hua, P.T. Dijke,·S. Kostidis, M. Giera, M. Hornsveld, TGFβ-Induced Metabolic Reprogramming during Epithelial-to-Mesenchymal Transition in Cancer. 77, 2103–2123 (2020). 10.1007/s00018-019-03398-6 10.1007/s00018-019-03398-6 PMC7256023 31822964

[62] J. Dudas, A. Ladanyi, J. Ingruber, T.B. Steinbichler, H. Riechelmann, Epithelial to Mesenchymal Transition: A Mechanism That Fuels Cancer Radio/Chemoresistance. Cells, 9 (2020). 10.3390/cells9020428 10.3390/cells9020428 PMC7072371 32059478

[63] A.D. Redfern, L.J. Spalding, E.W. Thompson, The Kraken Wakes: Induced EMT as a Driver of Tumour Aggression and Poor Outcome. 35, 285–308 (**2018)**. 10.1007/s10585-018-9906-x 10.1007/s10585-018-9906-x 29948647

