# Supplementary figures and images for: AKT-driven epithelial-mesenchymal transition is affected by copper bioavailability in HER2 negative breast cancer cells via a LOXL2-independent mechanism
Source: Cell Oncol (Dordr). 2022 Dec 1;46(1):93–115. doi: 10.1007/s13402-022-00738-w (PMC9947069; doi:10.1007/s13402-022-00738-w)

**a**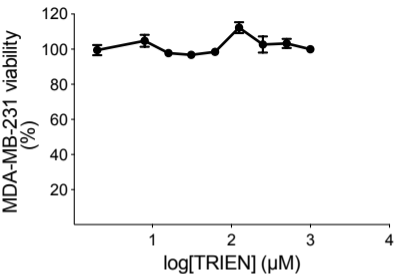**b**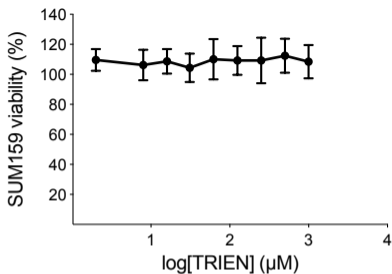**c**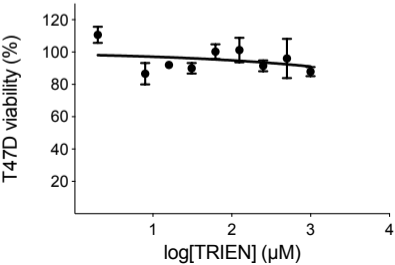**d**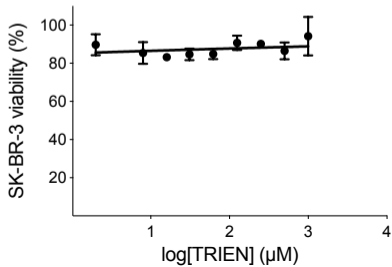

Supplement: Supplementary file 1 — Additional Figure 1 (PDF 388 KB) Effect of TRIEN treatment on breast cancer cell survival. Cells were exposed to different concentration of TRIEN (ranging from 2 to 1000 μM) for 72 h and cell viability assessed by MTS assay. (a) MDA-MB-231; (b) SUM 159; (c) T47D; (d) SK-BR-3. [file 13402_2022_738_MOESM1_ESM.pdf]

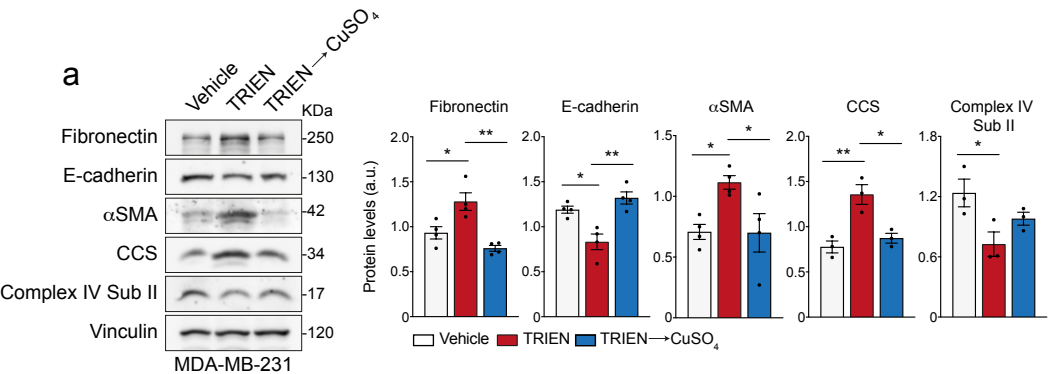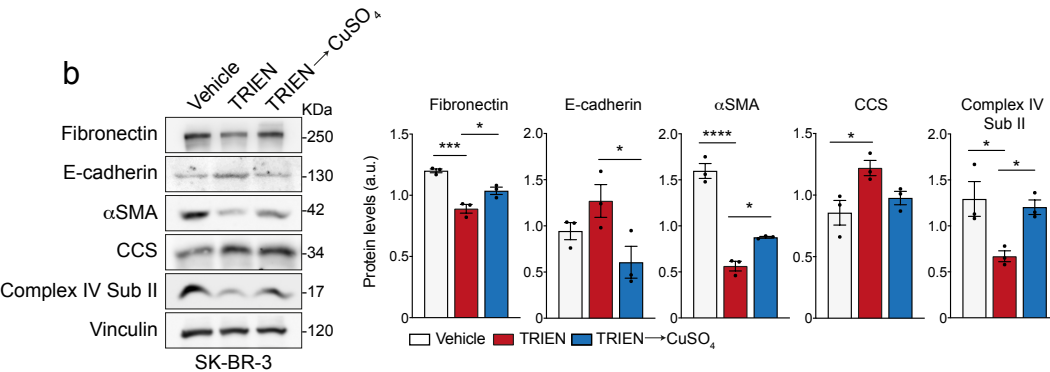

Supplement: Supplementary file 2 — Supplementary Figure 2 (PDF 730 KB) Restoring copper bioavailability reverts the altered expression of epithelial to mesenchymal markers. Cells were treated with 125 μM TRIEN for 24 (MDA-MB-231) or 48 h (SK-BR-3). Afterwards TRIEN was removed and cells incubated for 3 h with medium supplemented with 100 μM CuSO4. Western blot (left panels) and densitometric analyses (right panels) for fibronectin, E-cadherin, αSMA, CCS and the subunit II of cytochrome c oxidase are shown for (a) MDA-MB-231 and (b) SK-BR-3 cells. Twenty micrograms of proteins were loaded on each lane. Vinculin was used as loading control. One representative blot is shown for each antigen, data are presented as a mean ± SEM (n ≥ 3, ONE-way ANOVA *p < 0.05; ***p < 0.001 ****p < 0.0001). [file 13402_2022_738_MOESM2_ESM.pdf]

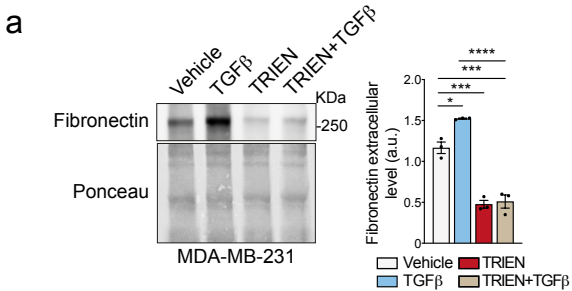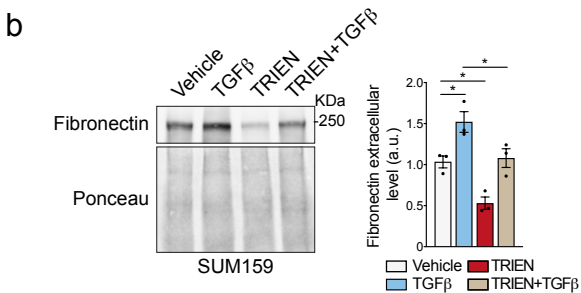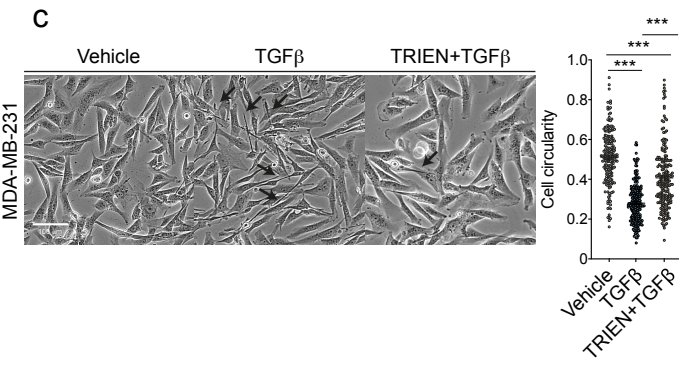

Supplement: Supplementary file 3 — Supplementary Figure 3 (PDF 5553 KB) Prolonged TRIEN exposure counteracts TGFβ treatment impairing the extracellular release of fibronectin and altering cells morphology. Western blot analysis of the level of extracellular release of fibronectin in (a) MDA-MB-231 and (b) SUM159, following 10 ng/ml TGFβ or 125 μM TRIEN treatment, for 24 h, alone and in combination. Ponceau S staining was used as loading control. (c, left panels) analysis of cell morphology and (c, right panels) cell circularity upon treatment with 10 ng/ml TGFβ alone or in combination with 125 μM TRIEN, for 24 h, were measured using the ImageJ software. One representative blot/image is shown; data are presented as a mean ± SEM (n ≥ 3, ONE-Way ANOVA, *p < 0.05, ***p < 0.001, ****p < 0.0001). Calibration bar corresponds to 100 μm. [file 13402_2022_738_MOESM3_ESM.pdf]

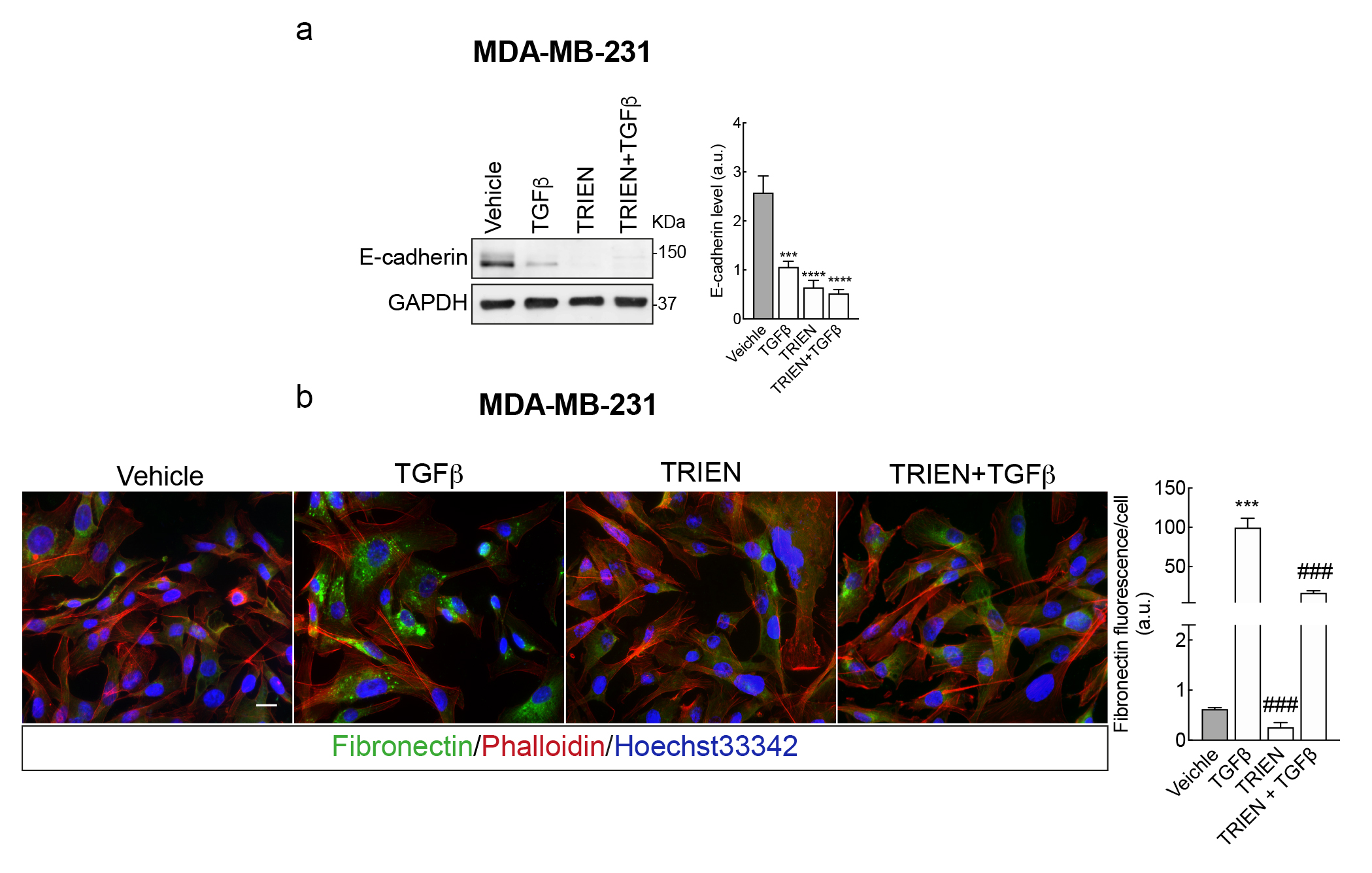

Supplement: Supplementary file 4 — Supplementary Figure 4 (JPG 973 KB) TRIEN pretreatment modulates TGFβ effects in MDA-MB-231 cells. a) Cells were treated with TGFβ (24 h) or 125 µM TRIEN (6 days), alone and in combination, Western blot (left panel) and densitometric analyses (right panel) for E-cadherin is shown. Twenty micrograms of proteins were loaded on each lane. GAPDH was used as loading control. One representative blot is shown for each antigen, data are presented as a mean ± SEM (n ≥ 3, ONE-way ANOVA, ***p < 0.001, ****p < 0.0001). b) Immunofluorescence images of fibronectin (left panels) and their corresponding signal analysis (right panel). Calibration bar: 100 μm. 40 × magnification. One representative image is shown; data are presented as a mean ± SEM (n ≥ 3, ONEway ANOVA, **p < 0.01 vs vehicle, ***p < 0.001 vs vehicle,###p < 0.001, vs TGFβ). [file 13402_2022_738_MOESM4_ESM.jpg]

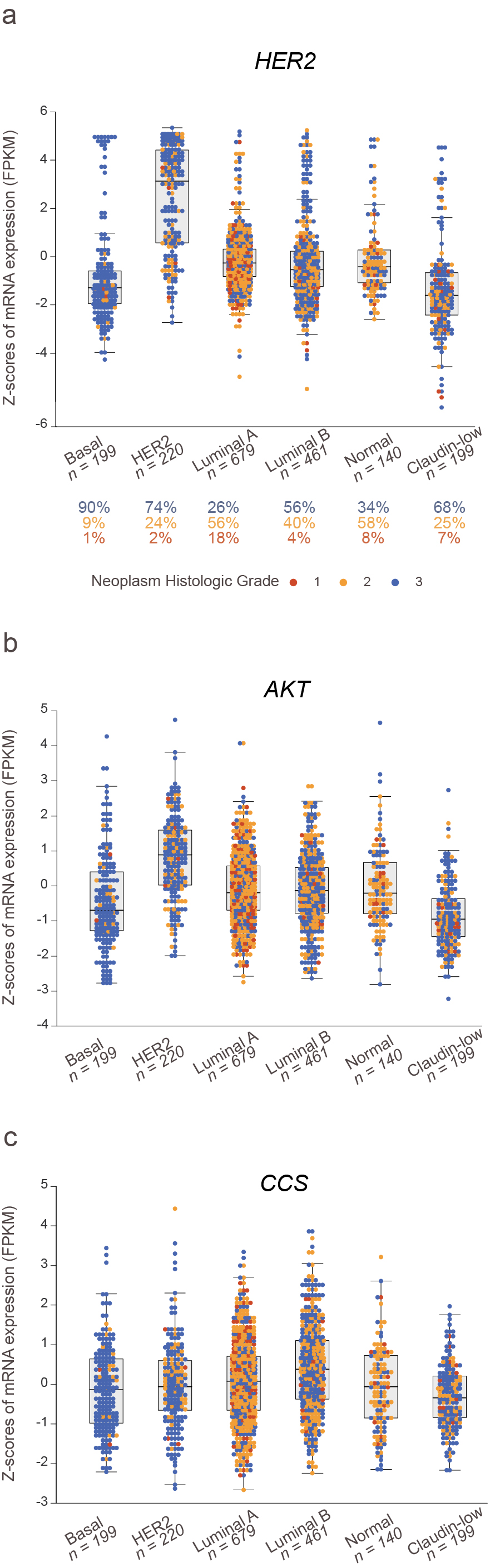

Supplement: Supplementary file 5 — Supplementary Figure 5 (JPG 891 KB) Claudin-low breast cancers are characterized by low levels of mRNA of HER2, AKT and CCS in comparison to other breast cancer subtypes. (a) HER2, (b) AKT and (c) CCS mRNAs expression levels across various classes of breast cancer were retrieved from The Cancer Genome Atlas Program (TCGA) database by the cBioPortal For Cancer Genomic tool. Dots in different colors represent the breast cancer histological grade of each patient included in the analysis: red for the first neoplasm histological grade, yellow for the second neoplasm histological grade and blue for the third neoplasm histological grade. In (a) the percentage of patients affected by the different histological grade is also reported for each subclass of breast cancer. [file 13402_2022_738_MOESM5_ESM.jpg]
